# Genetic diversity and characterization of rhinoviruses from Chinese clinical samples with a global perspective

**DOI:** 10.1128/spectrum.00840-23

**Published:** 2023-09-21

**Authors:** Peng Zhao, Nan Shao, Jie Dong, Haoxiang Su, Hongtao Sui, Ting Zhang, Fan Yang

**Affiliations:** 1 NHC Key Laboratory of Systems Biology of Pathogens, Institute of Pathogen Biology, Chinese Academy of Medical Sciences and Peking Union Medical College, Beijing, China; 2 Key Laboratory of Respiratory Disease Pathogenomics, Chinese Academy of Medical Sciences, Beijing, China; 3 State Key Laboratory of Respiratory Health and Multimorbidity, Beijing, China; U.S. Food and Drug Administration, Silver Spring, Maryland, USA

**Keywords:** Rhinovirus, genetic diversity, novel type, data mining, evolutionary dynamics

## Abstract

Rhinovirus (RV) is a significant pathogen causing upper and lower respiratory diseases and is one of the most prevalent human respiratory viruses. However, research on the genetic diversity and evolutionary dynamics of RVs in China and worldwide remains limited. To address this knowledge gap, we utilized Chinese clinical RV samples as a starting point and detected and reported 22 types, including A9, B3, and C13, in China for the first time. Among these, A67, A76, and A106 were also detected and reported in Asia for the first time, characterizing their genetic diversity. We also identified A110 as a novel type of RVA and reported its distinctive characteristics in phylogeny, secondary structure, and capsid protein structure. Furthermore, by mining, refining, and annotating RV sequence data available worldwide, four previously unreported novel types, B107, C58, C59, and C60, were identified, and their genetic diversity was revealed. Furthermore, we observed variations in the guanine-cytosine (GC) content among different serotypes and clades. Also of note was that, based on a complete and refined VP1 data set, the evolutionary dynamics of RV were analyzed systematically and on a large scale for the first time.

**IMPORTANCE:**

Based on clinical samples collected in China, we detected and reported 22 types for the first time in China, as well as three types for the first time in Asia, and reported their genetic characteristics and diversity. We identified a novel type of Rhinovirus (RV), A110, highlighting its unique genetic features. We annotated the genomic structure and serotype of all the existing RV sequences in the database, and four novel RV types were identified and their genetic diversity reported. Combined with the sequence annotation, we constructed a complete VP1 data set of RV and conducted the first large-scale evolutionary dynamics analysis of RV. Based on a high-quality data set, we conducted a comprehensive analysis of the guanine-cytosine (GC) content variations among serotypes of RVs. This study provides crucial theoretical support and valuable data for understanding RV’s genetic diversity and developing antiviral strategies.

## INTRODUCTION

Rhinovirus (RV) is a single-stranded, positive-sense RNA virus belonging to the family Picornaviridae, genus *Enterovirus*. The RNA genome is approximately 7.2 kb in length and is surrounded by an icosahedral protein capsid containing 60 structural proteins, surface-exposed VP1, VP2, VP3, and buried VP4 (1). Human is the exclusive host for RV, which can be categorized into “major” and “minor” groups based on the receptor used for host cell entry, namely, intercellular adhesion molecule-1 (ICAM-1) and low-density lipoprotein receptor (LDLR), respectively (2). Initially, RVs were divided into two groups based on the activity of antiviral drug combinations (3). Subsequently, the classification of RVs into two distinct species, RVA and RVB, was determined through analysis of viral sequences. The development of clinical molecular detection technology enabled the discovery of RVC, which cannot be cultured *in vitro* (4, 5). The receptor employed by RVC is the cadherin-related family member 3 receptor (6, 7).

RV is responsible for half to two-thirds of common colds and half of the exacerbations of asthma. In recent years, it has been reported that RV infection can also cause croup, bronchiolitis, community-acquired pneumonia, and other lower respiratory tract infections and aggravation of chronic lung diseases (8 – 12). RV takes a high economic toll when combining the costs of medical treatment, delayed education, work absenteeism, and missed economic and social development (13 – 17).

The prominent feature of RV is that it has a very large number of serotypes or types. Based on previous serological studies and molecular typing criteria based on VP1 and VP4/2 *p*-distance (18, 19), RVA has 80 types, RVB has 32 types, and RVC has 57 types. This diversity of RVs has hindered drug discovery efforts. Similarly, the development of vaccines against RV has encountered comparable challenges. Despite numerous attempts, no protective vaccine has been approved, although several are currently in various stages of development (20); mainly due to the 169 types of RV, the vaccine is difficult to generate cross-serotype immunity (21). At the same time, it also poses a challenge to the archiving, management, annotation, and accurate typing of RV data.

The GC content of a viral genome is known to be closely associated with various factors, including genome stability, adaptability for virus survival, amino acid composition, and transcription and translation efficiency. The investigation of GC content in viruses serves as a significant indicator for understanding their genetic diversity. Previous studies have indeed reported that the genome of RV exhibits a lower GC content compared to genomes of other enteroviruses. Additionally, among the RV serotypes, it has been observed that RVC exhibits the highest GC content (5, 22, 23). However, due to the vast number of RV serotypes and the limited availability of thoroughly curated and annotated high-quality RV sequences as fundamental research resources, a research gap still exists regarding whether there are differences in GC content among different serotypes.

At present, there are few studies and reports on RV in China, and there is also not much investment in RV detection and scientific research globally. However, the severe respiratory-related illnesses caused by SARS, MERS, and SARS-CoV-2 have demonstrated the enormous impact respiratory viruses can have on human society (24 – 26). When a virus, particularly an RNA virus with a high mutation rate, has not led to significant public health crises, it is crucial to enhance the investigation of its genomics, genetic diversity, and evolutionary dynamics. This endeavor allows us to deepen our scientific understanding of the virus and effectively mitigate the social impact and health risks associated with virus-related diseases.

To address the aforementioned research gaps, we collected and detected clinical RV samples in China and reported their genetic diversity and characteristics by combining phylogeny, virus recombination analysis, and other perspectives or methods. There are many serotypes of RVs, and a significant portion of the virus typing information in the GenBank database is inaccurate or incomplete. In order to overcome the challenge, we performed typing annotations on all untyped sequences in the GenBank database and identified four previously unreported RV types. Based on the complete data set we constructed, it was observed that the GC content of the RV genome varies not only among different serotypes but also within different clades. Moreover, through the integration of evolutionary dynamics analysis, the phylogenetic history of RVs and population changes over time scales were revealed for the first time. Collectively, starting from the clinical samples of RV in China, the present study provided important theoretical support and foundation for the research on the genetic diversity and evolutionary dynamics of RV in China, Asia, and the world. It also provided academic support and theoretical motivation for the prevention and treatment of RV-related diseases and the development of antiviral strategies.

## RESULTS

### Clinical sample sequencing and identity analysis

**Fig 1:**
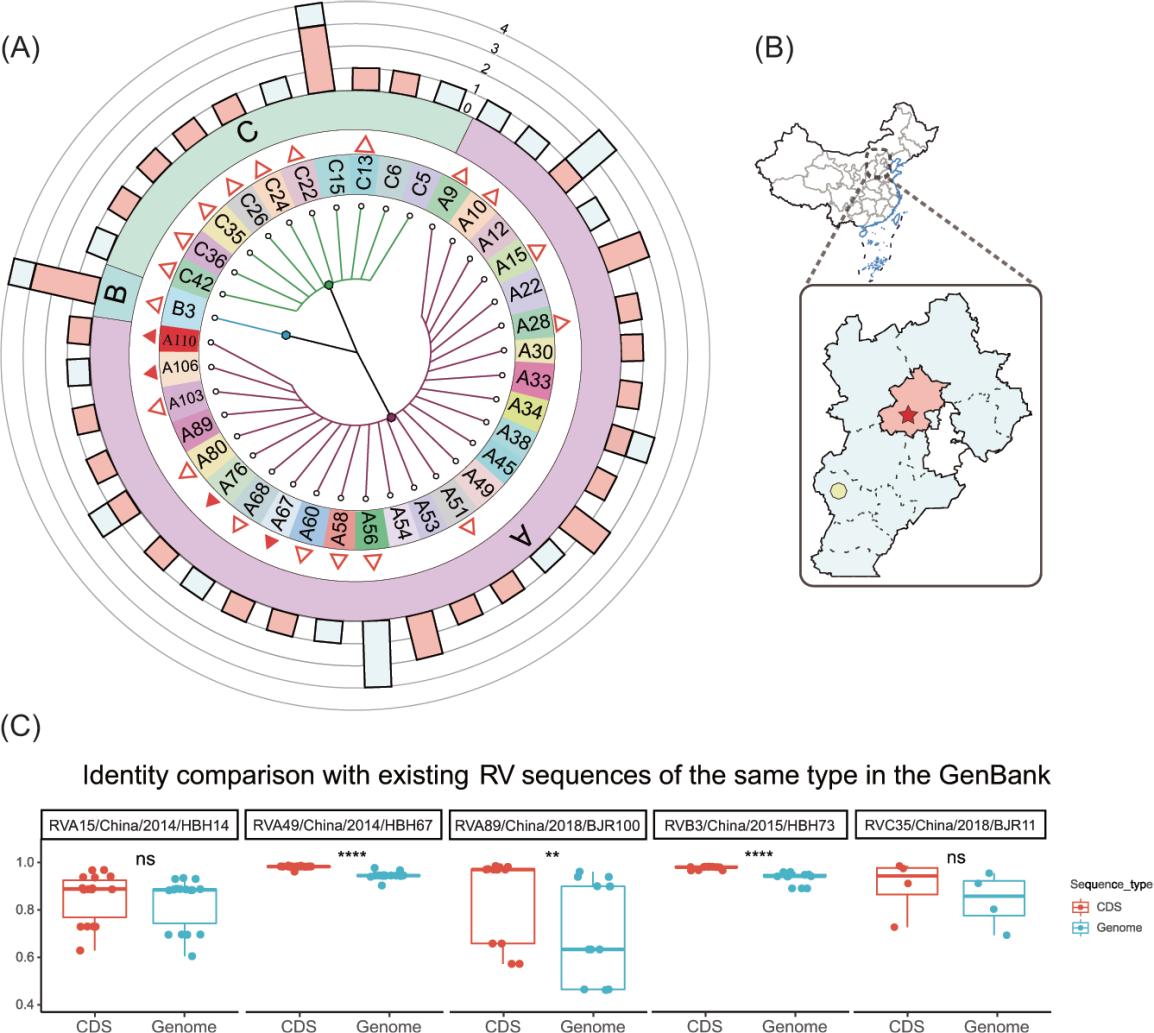
The information of sampling and RV sequences identities. (**A**) A taxonomic tree of RV types collected and sequenced in this study. The solid and non-solid red triangles on the taxonomic tree represent the first detection and report of this RV type in Asia and China, respectively, and A110 is a new type of RVA. The multi-value bar chart in the outermost ring represents the number of virus sequences detected by the corresponding type (red and blue represent samples collected in Beijing and Hebei Province, respectively). (**B**) A map with the locations of sample collection, the red five-pointed star means Beijing Children’s Hospital Affiliated to Capital Medical University, and the yellow octagon represents Hebei Children’s Hospital, which corresponds to the content in **A**. (**C**) A boxplot of the identity comparison between the RV genome nucleic acid sequence and the AA sequence deduced by CDS of partial samples and all isotype RVs in the GenBank database. See Fig. S1 for full.

In this study, between 2014 and 2018, 52 RV-positive samples were detected from 209 clinical samples collected at Children’s Hospital Affiliated to Capital Medical University in Beijing, China, and 82 clinical samples collected at Hebei Children’s Hospital in Hebei Province, China (Table S1). After sequencing and data analysis, the specific sample collection locations and the distribution of RV types are shown (Fig. 1A and B).

It is worth noting that as of 12 May 2022, based on the “collection_date,” “country,” and other field information in the GenBank database, three types of RVA, A67, A76, and A106, were detected and reported for the first time in Asia. Another 11 types, including A9, A10, A15, A28, A51, A56, A58, A60, A68, A80, and A103, were detected and reported for the first time in China. B3 in RVB was detected and reported for the first time in China. Seven types of RVC, including C13, C22, C24, C26, C35, C36, and C42, were detected and reported for the first time in China. Furthermore, we detected a novel type of RVA: A110.

We performed pairwise identity comparison analysis of 52 RV genomic nucleic acid sequences and deduced coding sequences (CDS) amino acid (AA) sequences with all existing isotype sequences in the GenBank database. The box plot results indicated that the majority of sequences, both in terms of nucleic acid and amino acid sequences, exhibited no significant differences compared to the existing RV-isotype sequences in the database (Fig. S1). However, the pairwise identity of A15 (genome range, 61.4%–94.4%; CDS range, 63.9%–97.8%), A89 (genome range, 47.4%–97.2%; CDS range, 58.4%–99.5%), C35 (genome range, 68.5%–95.6%; CDS range, 72.0%–98.7%) was significantly different compared to the isotype sequences in GenBank (Fig. 1C).

### Phylogenetic and recombination analysis

Genome-based maximum likelihood (ML) phylogenetic trees of RVA, RVB, and RVC were constructed from the RV sequences detected in clinical samples, the highly homologous homotypic sequences in the database, and the prototype strain sequences of all types, in which the red label font represented the RVs detected in this study.

**Fig 2:**
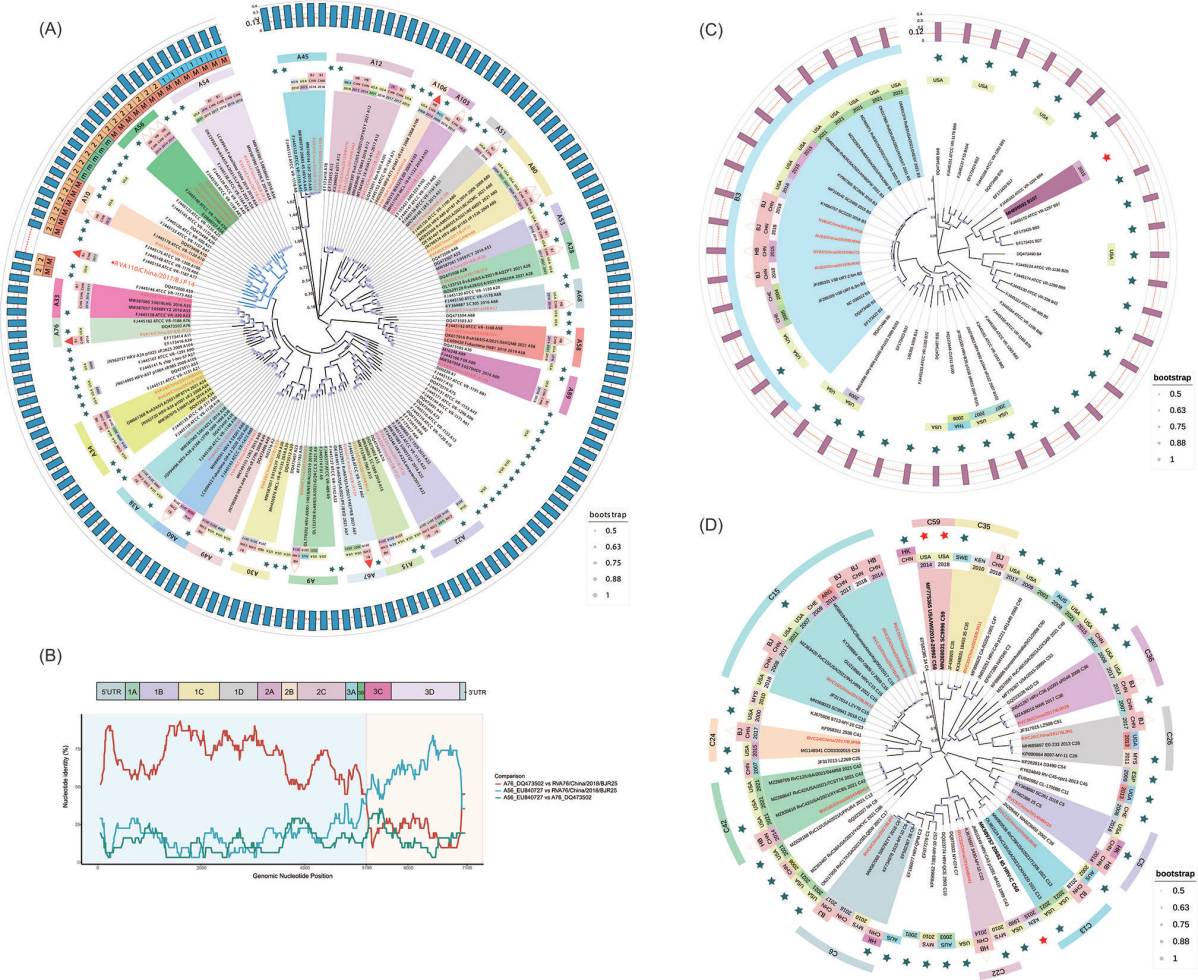
Phylogenetic and recombination analysis of RVs. (**A, C, D**) Genome-based phylogenetic trees of RVA, RVB, and RVC, respectively. The label text above the phylogenetic tree is in red, indicating the RV present study sequenced. Others include isotype RVs screened by BLAST and prototype strains. Their label text includes accession number in GenBank, virus strain name, and serotype. The outer ring represents the sampling year, the ISO3 code for sampling country, the Chinese province or city, the novel type RVs, the prototype strain information (green five-pointed star represents the prototype strain, red five-pointed star represents the discovered novel type RV, and solid and non-solid triangles represent whether it was first detected and reported in Asia or China), and serotype strip. The outside of the serotype strip in (**A**) contains receptor information [the major (**M**) ICAM-1 receptor or the minor (**M**) LDLR receptor] and activity information of antiviral compounds (group “1” or group “2”). The outermost rings of (**A **and **C**) are simple bar charts, which represent the VP1 *p*-distance of RVA110 and RVB107 to the prototype strain and others, respectively. (**B**) A representative recombination result of RVA76/China/2018/BJR25 as the recombinant sequence, DQ473502 as the minor parent, and EU840727 as the major parent, which is shown by the pairwise identities chart.

The RVA genome phylogenetic tree systematically displayed the phylogenetic relationships of 35 RVA sequences within and between types (Fig. 2A). It is worth noting that RVA12/China/2014/HBH43 and RVA12/China/2015/HBH70 collected in Hebei in 2014 and 2015 are closer to the phylogeny of RV collected in Mexico in 2010, while the RVA12/China/2017/BJP66 collected in Beijing in 2017 is more similar to the sequences collected in Jiangxi Province in the same year, and they are in two clades (bootstrap value equal to 1) with the aforementioned sequences from Mexico and Hebei.

**Fig 3:**
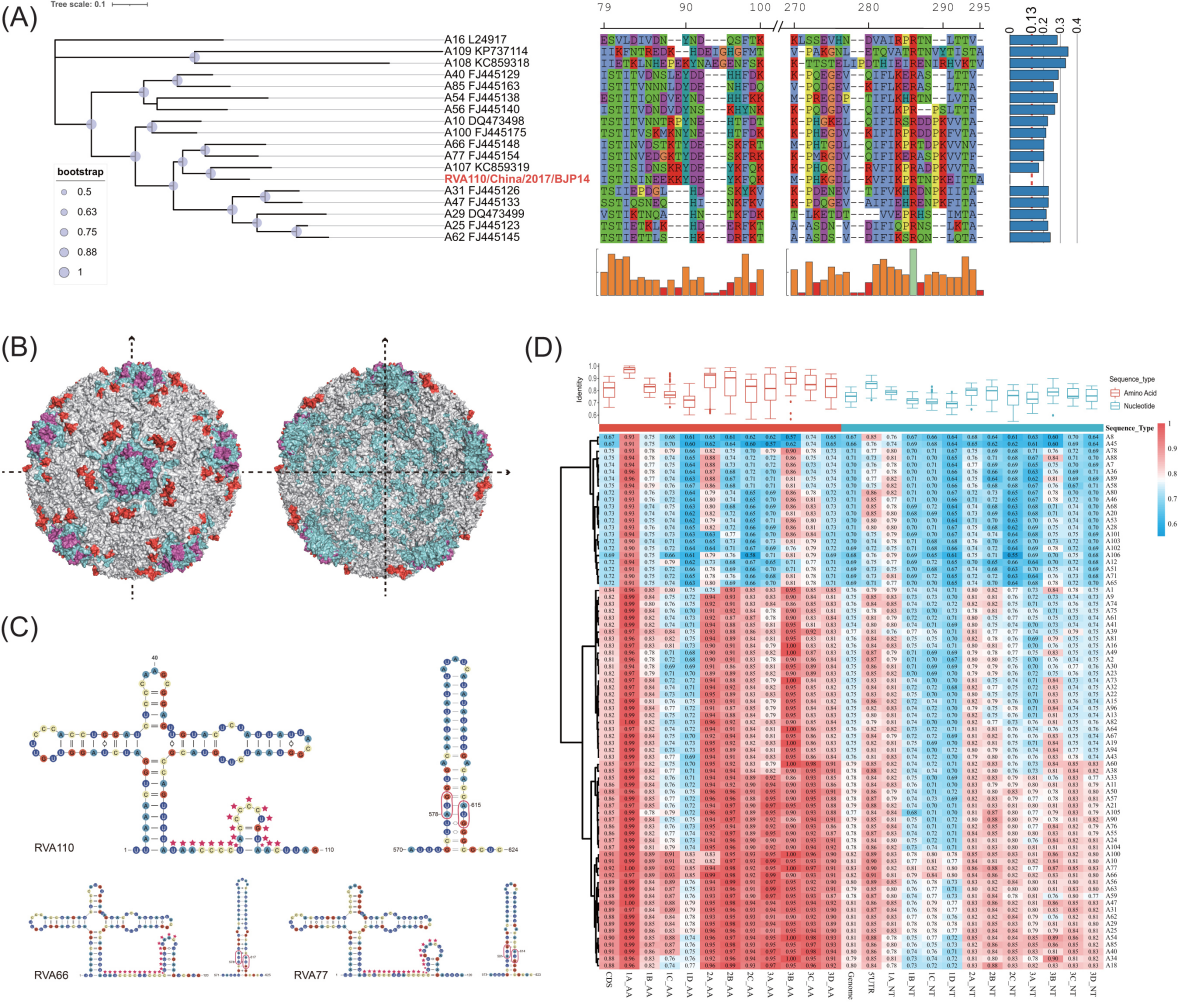
Genetic diversity of RVA110. (**A**) The VP1 phylogenetic tree; the right side is the multiple sequence alignment display of the two HRVs of the corresponding VP1 protein (the bottom is the corresponding conservation bar of the alignment). On the far right is a bar chart of VP1 *p*-distance for RVA110 versus other sequences. The complete external capsid structure (left) and profile (right) of RVA16 (GenBank accession: L24917, PDB ID: 1AYM) are shown in (**B**); in addition, VP1 is marked in blue, and its HRV1 and HRV2 are marked in purple and red, respectively. (**C**) The predicted 5’UTR secondary structures of RVA110, RVA66, and RVA77; the area marked by the red pentacle is the pyrimidine-rich spacer segment. (**D**) A pairwise identities fusion plot of the heat map and box plot (RVA107, RVA108, and RVA109 not included).

The RVB genome phylogenetic tree systematically showed the phylogenetic relationships of four RVB sequences within and between types (Fig. 2C). The phylogenetic relationship of the four B3 viruses was closer to the B3 reported in Switzerland in 2004 and 2005.

The RVC genome phylogenetic tree systematically showed the phylogenetic relationships of 13 RVC sequences within and between types (Fig. 2D). It is worth noting that RVC15/China/2017/BJR32 clustered with three RV strains (RVC15/China/2014/HBH30, RVC15/China/2018/BJR73 in the same region, and RVC15/China/2017/BJP1 in the same region and year) in two phylogenetic clades (bootstrap value equal to 1).

All the above viral sequences were used to construct a recombination analysis data set, and after multiple sequence alignments, recombination events were detected by RDP (27). We considered recombination events detected simultaneously by three or more programs with a median *P*-value of less than 0.00001 to be reliable. As a result, a total of seven recombination events were detected (Table S3). Among them, we unexpectedly found that RVA76/China/2018/BJR25 may be recombined from DQ473502 (A76, minor parental sequence) and EU840727 (A56, major parental sequence), and the recombination breakpoint located at 3C (Fig. 2B).

### A110 has unique genetic characteristics

A110, collected from Beijing, China, on 3 March 2017, was a novel type RVA, which belongs to the same lineage as A77 and A66 in genome phylogeny and has obvious phylogenetic differences (bootstrap value is equal to 1). The VP1 *p*-distance between the A110 and RVA prototype strains of 80 types was greater than the typing threshold of 0.13 (19), as shown in the outermost bar chart (Fig. 2A and 3A). Because A107, A108, and A109 do not have complete genome sequences (Table S2), to interpret the phylogenetic relationship and evolutionary history of A110 more comprehensively, we constructed a VP1-based phylogenetic tree for 13 prototype strains that were located in the same clade as A110 (Fig. 2A and 3A), A107, A108, A109, and A16, among which A16 has tertiary capsid protein structure. We saw that A107 was closest to A110 in the phylogenetic tree constructed based on VP1 (Fig. 3A).

Through multiple sequence alignment of VP1 AA sequences, we found two hypervariable regions (HVR1:79–100 and HRV2:270–295) (Fig. 3A; Fig. S2A). Studies have hypothesized that the HRV1 region was the neutralizing immunogenic site (NIm) of A16 (28). In addition, A110 had an alanine appended to the end of the HVR2. Although the function of HVR2 has not been reported yet, we found that both HVRs were located on the surface of the RV capsid protein (Fig. 3B). Therefore, we hypothesized that HVR2 may also be an important NIm.

Previous studies have found that RVs have the same 5′-terminal cloverleaf-like motifs (CLs), a pyrimidine-rich spacer segment, and a bait-and-switch structure as all enteroviruses. They may be helpful for the initiation of RNA synthesis and help convert infecting genomes from translation to replication template (29). We predicted the RNA secondary structures of A110, A66, and A77 and found that A110 also had CLs, a pyrimidine-rich spacer segment, and bait-and-switch structure like other RVs. In contrast to A66 and A77, A110 exhibited an early emergence of a small stem-loop structure within the pyrimidine-rich spacer segment (Fig. 3C).

To display the genetic diversity of A110 in a more systematic, comprehensive, and multidimensional way, we designed a fusion chart of boxplot and cluster heat map to show the pairwise identity matrix and distribution more specifically by calculating the pairwise identities of each genome fragment of A110 and all other RVA types (except A107, A108, and A109). The heat map clustering pattern was roughly the same as the phylogenetic relationship (Fig. 2A and 3D).

### Data mining for novel RVs

**Fig 4:**
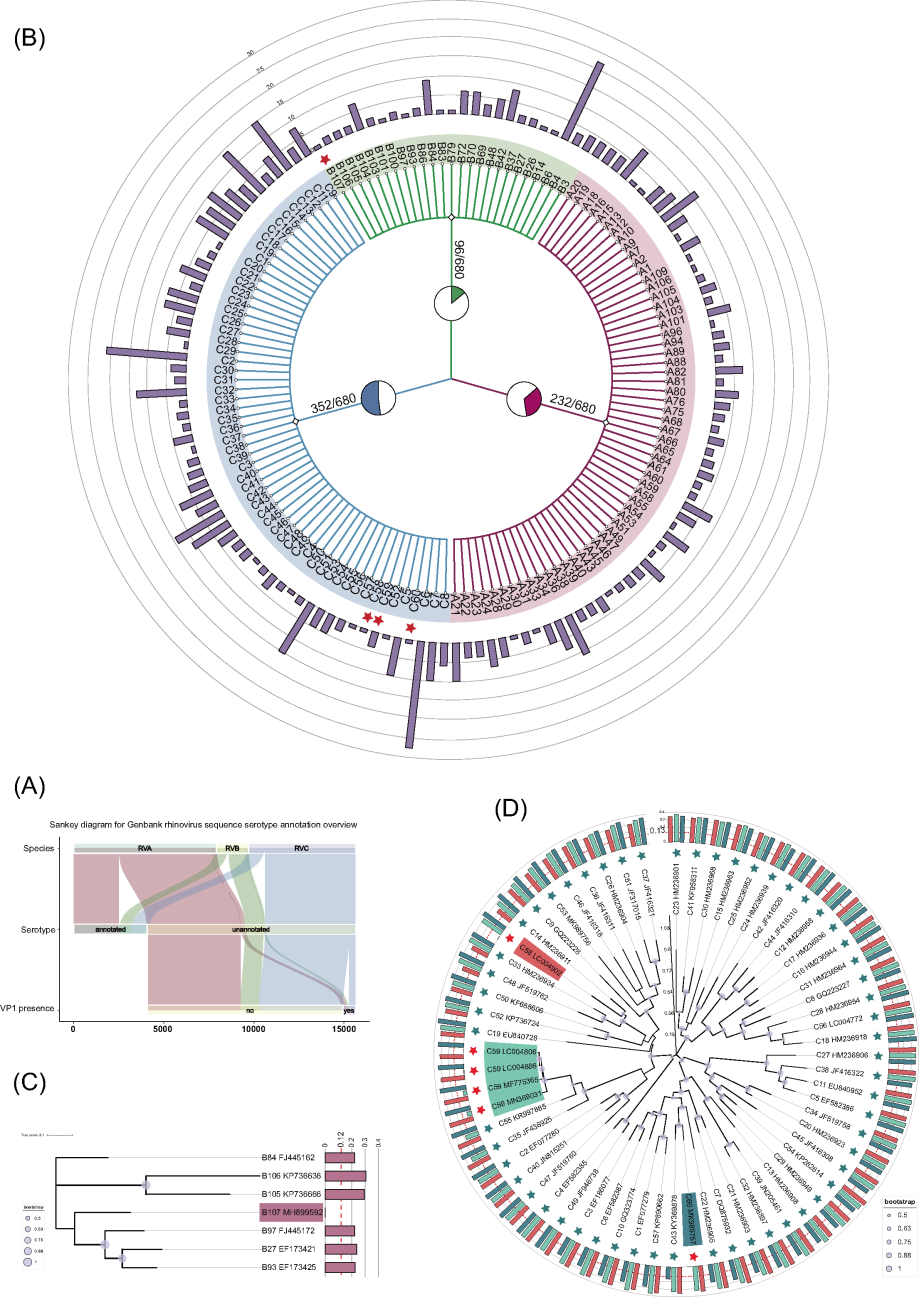
Data mining, typing annotation, and phylogenetic relationships of novel RVs. (**A**) A sankey diagram for GenBank RV sequence type annotation result overview. (**B**) A species taxonomic tree shows the RV typing result, and the pie chart on the branch represents the proportion of RV type distribution. The red five-pointed star represents the novel type RVs (B107, C58, C59, and C60), and the bar chart in the outer ring represents the quantity. (**C **and **D**) The VP1 phylogenetic trees of RVB and RVC, respectively. The multi-valued bar graph in (**D**) represents the VP1 *p*-distance between novel type RVC [C58, C59 (LC004806), and C60 from left to right] and prototype strains.

As of 12 May 2022, GenBank has included nucleic acid sequences RVA7921, RVB1687, and RVC5921. After data screening, 11,779 sequences without type annotation (the Organism field is “Rhinovirus A,” “Rhinovirus B,” or “Rhinovirus C”) were found, including 5,651 RVAs, 1,120 RVBs, and 5,008 RVCs, respectively. VP1 annotating was performed on all unannotated sequences, among which 680 had VP1 sequences with completeness greater than 90%. *P*-distance was calculated by comparing with each type of prototype strain for RV typing (Fig. 4A; Table S4). The annotation results showed that for 232 RVA sequences, typing matched 59 types, such as A1, A2, and A7. For 96 RVB sequences, typing matched 25 types, such as B3, B4, and B6, among which B107 was a novel type RVB. For 352 RVC sequences, typing matched 59 types, such as C1, C2, and C3, among which C58, C59, and C60 were novel (Fig. 4B).

The outermost bar chart represents the VP1 *p*-distance of each type of prototype strain and B107. It can be seen that the VP1 *p*-distance of the other 32 types and B107 were all greater than the typing threshold of 0.12 (19). On the whole-genome phylogenetic tree, B107 was more similar to B97, B93, and B27 (Fig. 2C), and on the VP1 phylogenetic tree, which included B105 and B106 without a complete genome sequence, the phylogenetic relationship was the same as that on the genome phylogenetic tree (Fig. 4C).

Because there is no complete genome sequence of C58, C59 and C35 were located in a clade on the whole-genome phylogenetic tree, and C60 and C43 were located in a clade, and the bootstrap value of the corresponding node is 1 (Fig. 4D). However, in the phylogenetic tree constructed based on VP1, all types of RVC exist. There, C58 was phylogenetically most closely related to C14. C59 was closest to C55, followed by C35. C60 was closest to C43. This was the same as that shown in the genome phylogenetic tree. The most lateral multi-value bar chart represents the VP1 *p*-distance between each type of prototype strain and C58, C59, and C60 (Fig. 4D), which all exceeded the RVC typing threshold 0.13 (18, 19).

### RV differ in GC content among serotypes

Based on the aforementioned work, we have acquired a comprehensive and refined initial data set comprising serotype-annotated RV sequences. To conduct a more rigorous comparison of the potential differences in GC content among RV serotypes, we performed a screening process to eliminate low-quality sequences. Subsequently, we constructed a high-quality RV genome data set. The average GC content for each serotype was calculated and recorded in the Table S5.

**Fig 5:**
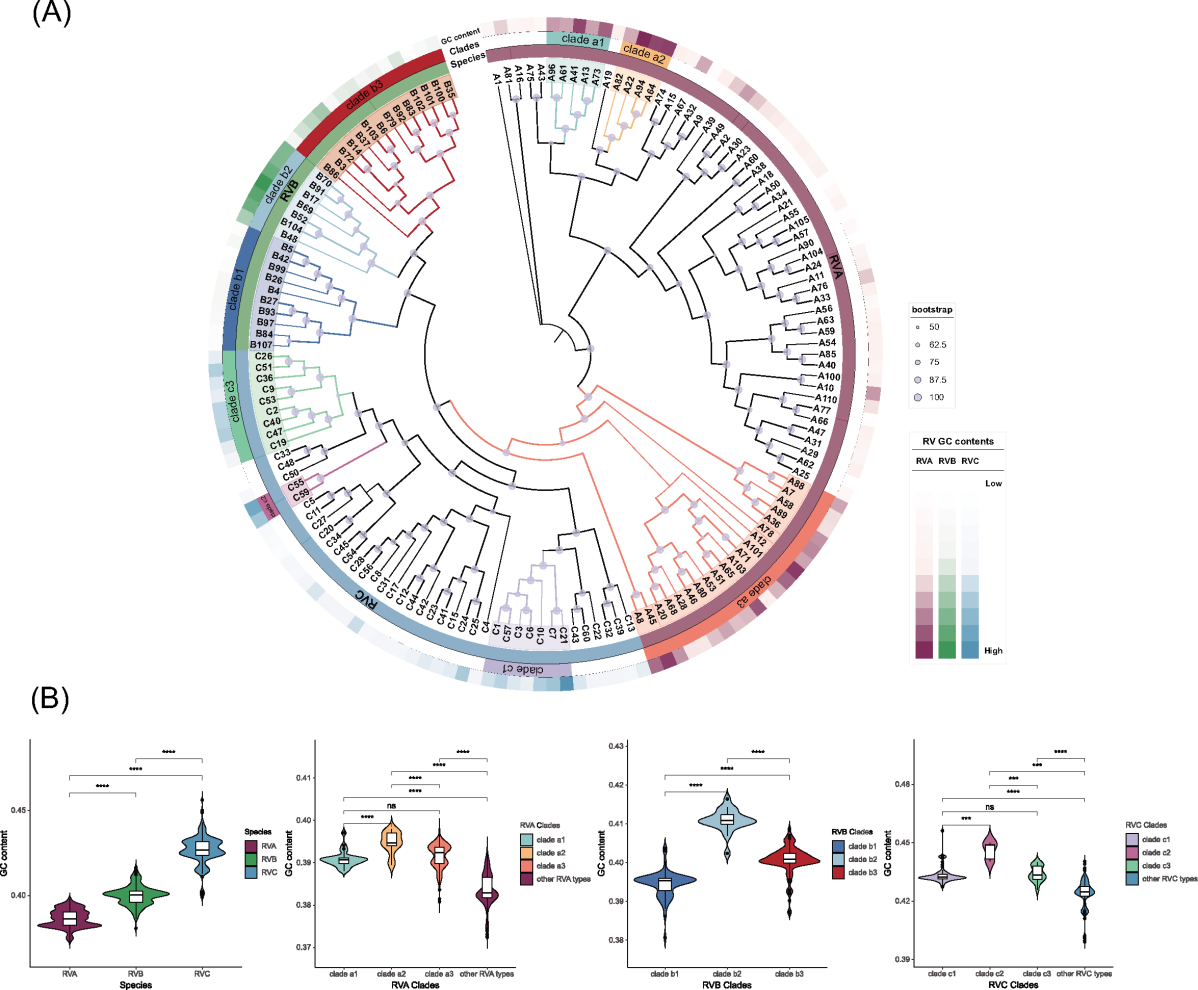
GC content comparison between RV serotypes and clades. (**A**) The phylogenetic tree of serotypes with high-quality complete genome sequences is constructed based on the whole genome. The outer rings of the tree represent the species, followed by the clades. The outermost heatmap displays the normalized GC content data of different serotypes within their respective species (RVA, RVB, and RVC represented by three different colors). (**B**) Violin plots comparing the GC content between different species and clades, corresponding to the colors in (**A**).

The GC content of RVA is found to be lower compared to RVB, whereas RVC exhibits the highest GC content (Fig. 5B). These findings are consistent with a previous study (22, 23). To facilitate a more comprehensive comparison of the GC content differences among serotypes, we performed normalization by calculating the relative GC content within each species. The results are visualized in the outermost ring of the RV phylogenetic tree as a heat map (Fig. 5A).

Significant variations in GC content were observed among different serotypes of RV, even within the same species. Moreover, these differences in GC content were found to be associated with the phylogenetic relationships among the serotypes.

As depicted in Fig. 5 and the Table S5, among RVA serotypes, A22 exhibited the highest GC content (0.3963), while A104 displayed the lowest GC content (0.3751). Notably, we identified three clades (clade a1, clade a2, and clade a3, excluding RVB and RVC branches) with higher GC content. Among these, clade a2 exhibited a significantly higher GC content compared to other clades and serotypes. Within RVB, B69 exhibited the highest GC content (0.4164), while B99 had the lowest GC content (0.3862). Distinct differences in GC content were observed among the three RVB clades, with clade b1 having the lowest GC content and clade b2 having the highest. In the case of RVC, C21 displayed the highest GC content (0.4562), whereas C48 exhibited the lowest GC content (0.4007). Additionally, we identified several clades with higher GC content in RVC, among which clade c2 showed a significantly higher GC content compared to clade c1, clade c3, and other RVC serotypes.

### Evolutionary dynamic analysis of RVA, RVB, and RVC

We obtained a relatively comprehensive RV VP1 data set by conducting sample sequencing, data mining, and type annotation. This data set encompassed a greater variety of RV types compared to the whole genome data set, making it suitable for analyzing the evolutionary dynamics. First, we used the Bayesian evaluation of temporal signal (BETS) method to evaluate the temporal signals on the three datasets of RVA, RVB, and RVC. Their log Bayes factors were all greater than 5, which indicated that they all had strong temporal signals (Table 1).

**TABLE 1:** VP1 data set size and BETS result for evolutionary dynamic analysis

	RVA	RVB	RVC
Data set size (sequence number)	1,078	223	687
Log[*P*(Y|*M*_iso_)]	−69,311.76	−22,167.05	−68,349.10
Log[*P*(Y|*M*_het_)]	−68,713.61	−22,083.36	−67,848.41
Log Bayes factor	598.15	83.69	500.69
Temporal signal	√	√	√

**Fig 6:**
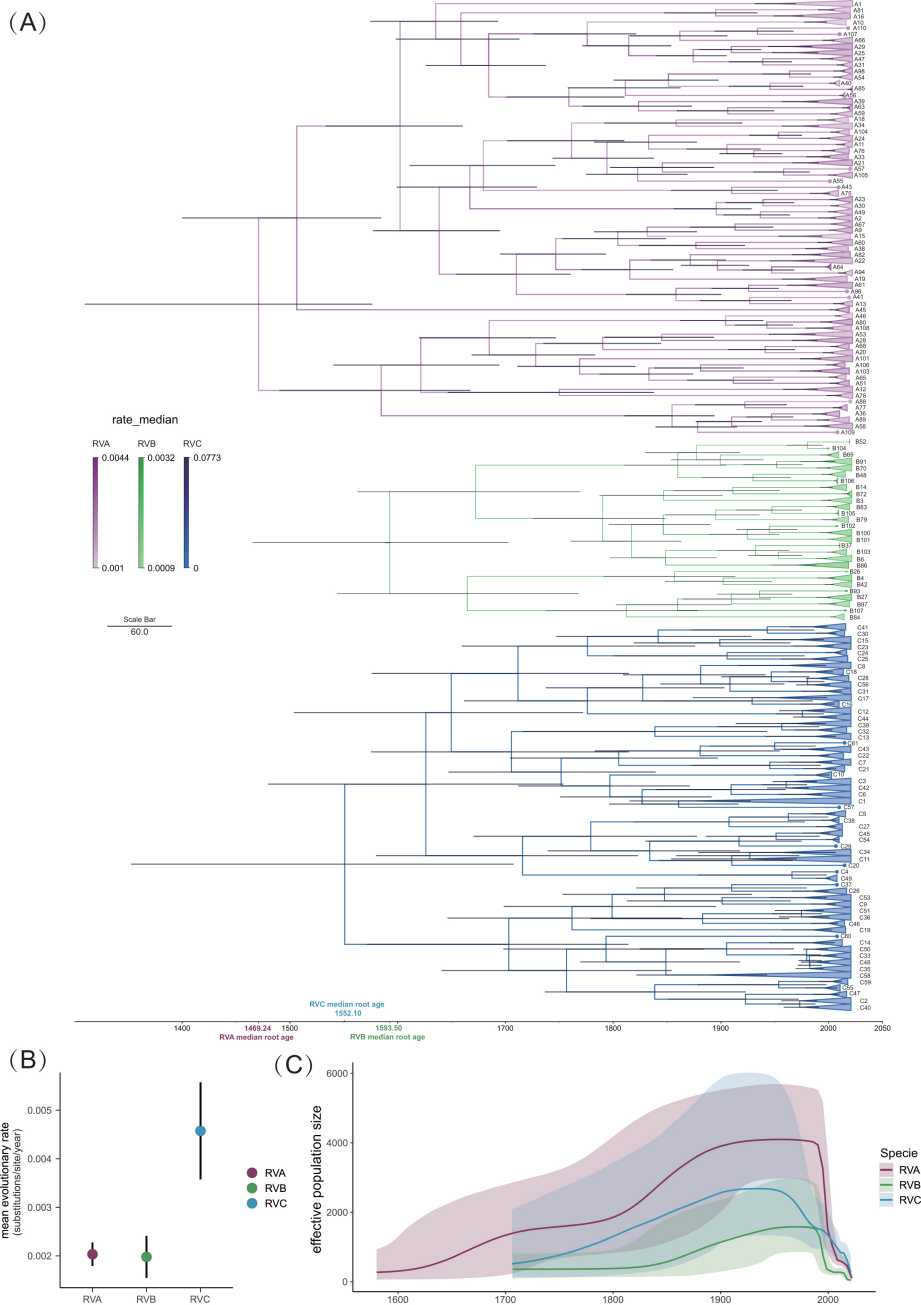
Demographic history reconstruction and evolutionary dynamic analysis of RVA, RVB, and RVC. (**A**) Time-scaled MCC trees of the VP1 gene of RVA, RVB, and RVC constructed using BEAST (version 1.10.5). The rate median of the branches of the three trees is indicated by the shade of color. To make the presentation more intuitive, RVs of the same type located in the same clade are collapsed into triangles. (**B**) The mean evolutionary rate of RVA, RVB, and RVC, with the black line at the top and bottom representing the 95% HPD. (**C**) Population dynamic analysis of RVA, RVB, and RVC, inferred via a Bayesian skyline coalescent tree prior. The intervals represent 95% HPD of the product of generation time and effective population size *N*_e_ (**T**). The middle line tracks the inferred mean of *N*_e_ (**T**).

The phylogenetic relationships of types between the VP1-based, time-scaled maximum clade credibility (MCC) trees of RVA, RVB, and RVC were similar to those of the genome-wide, VP1-based ML phylogenetic trees (Fig. 6A, 2A, C, and D, 3A, 4C and D). In addition, the MCC tree revealed the most recent common ancestor (tMRCA) and the time to the tMRCA for RVA, RVB, and RVC and their serotypes or types. We estimated tMRCA for RVA at 1469.24 with a 95% highest posterior density (HPD) range of (1307.83–1574.61). The estimated tMRCA was 1593.50 (1467.76–1703.55) for RVB and 1552.10 (1351.24–1706.12) for RVC (Fig. 6A).

As shown in Fig. 6B, the mean evolutionary rate was estimated at 2.086 (1.769–2.244) × 10^−3^ substitutions/site/year for RVA, 1.997 (1.536–2.402) × 10^−3^ substitutions/site/year for RVB, and 4.560 (3.582–5.582) × 10^−3^ substitutions/site/year for RVC. The color shades of the branches of the three MCC trees represent different median evolutionary rates. A64 (2.94 × 10^−3^), A103 (2.58 × 10^−3^), B86 (2.66 × 10^−3^), B14 (2.40 × 10^−3^), C15 (5.58 × 10^−3^), and C9 (4.72 × 10^−3^) had higher evolutionary rates. A46 (1.35 × 10^−3^), A15 (1.35 × 10^−3^), B79 (1.53 × 10^−3^), B84 (1.53 × 10^−3^), C44 (0.16 × 10^−3^), and C33 (0.35 × 10^−3^) had lower evolutionary rates. The phylogenetic relationships of the 52 RV samples reported in this study are shown in non-collapsed MCC trees by FigTree software (Fig. S3 to S5).

As shown in Fig. 6C, the estimated effective population sizes *N*_e_(t) over time of RVA, RVB, and RVC indicated that the effective population size and relative genetic diversity of the three RVs have exhibited a consistent upward trend since their initial emergence, reaching their peak during the period of 1950–2000. However, this peak was subsequently followed by a rapid decline. Furthermore, RVA exhibited the highest effective population size, followed by RVC, while RVB demonstrated the lowest effective population size. However, after 2000, the effective population size of RVC and RVA showed a gradual convergence.

## DISCUSSION

RVs are one of the most prevalent human respiratory viruses and a common cause of influenza-like illness (30, 31). However, there are few systematic studies on its genetic diversity, genomic characteristics, and evolutionary dynamics. In this study, we sequenced clinical RV samples collected in China to obtain their genome sequences. Referring to the sequence meta-information in the GenBank, we identified and documented some novel RV types, previously unreported in China and Asia (Fig. 1A and B). The first-time detection of these RV types may be attributed to new transmission patterns; however, it could also be indicative of lack of information due to the limited research done in RVs. The aim of this study was to contribute to fill this gap. Our findings revealed minimal differences in nucleic acid and amino acid sequences between the RV sequences obtained from our samples and those of the same serotype published in the database (Fig. S1). However, A15, A89, and C35 showed substantial differences and identity changes (Fig. 1C); thus, monitoring of RV diversity and clinical manifestations is warranted to determine whether these distinct viruses acquired new trains.

Phylogenetic and recombination analyses of these RV sequences can help us understand their phylogenetic relationships and evolutionary history. The ML phylogenetic trees of RVA, RVB, and RVC showed that RVs could spread and circulate globally across regions, countries, and continents (Fig. 2A, C, and D). Among them, A89, A53, A54, A33, A38, A30, C6, and C5 all had a close phylogenetic relationship with the RVs reported in China. This suggested that they may originated from domestic epidemics. The sequences of RVA12/China/2014/HBH43 and RVA12/China/2015/HBH70 collected in Hebei in 2014 and 2015 were closer to those collected in Mexico in 2010. However, the RVA12/China/2017/BJP66 sequence collected in Beijing in 2017 was more similar to the sequence collected in Jiangxi Province, China, in the same year, indicating that A12 may had different evolutionary origins in different regions of China (Fig. 2A; Fig. S3). According to the MCC tree of RVB, three strains in Beijing in 2018 and one strain in Hebei Province in 2015 were located in two clades. The tMRCA of them was 2014. We speculate that B3 in Beijing was transmitted from Hebei Province (Fig. S4). RVC15/China/2017/BJR32 collected in Beijing was located in the same clade as RVC15/China/2014/HBH30 collected in Hebei. RVC15/China/2018/BJR73 collected in Beijing was in a different clade, together with RVC15/China/2017/BJP1 collected in the same region and year (Fig. 2D; Fig. S5). This suggested that C15 circulating in the same area and year could still have different evolutionary sources.

Recombination has significantly contributed to the diversification of RNA viruses, leading to the emergence of novel isolates, lineages, species, and occasionally, even new viral families (32 – 34). Virus recombination also plays a significant role in the evolution and shaping of genetic architecture, particularly observed in enterovirus, such as Entreovirus B (EVB), which undergo frequent recombination events (35). In contrast, RV have a low recombination frequency compared to other enteroviruses. While there are 169 serotypes of RV, point mutations are more influential than recombination in the process of virus evolution and the formation of genetic diversity.

An important study has evaluated the recombination of RVs and identified some recombination events between types (29). Previous analysis showed that there was little evidence of recombination between RVB and RVC in the coding region, whereas the RVA sequence appeared to undergo numerous recombination events (36). Correspondingly, seven recombination events were detected by more stringent screening criteria, including five RVAs, one RVB, and one RVC, and no recombination events were detected between different species (Table S3). In addition, it is worth noting that the earliest reported primary recombinant sequence for the majority of these recombination events was not detected within our RV samples. However, an intriguing finding emerged with the identification of RVA76/China/2018/BJR25 as a prominent recombinant sequence. This sequence displayed a distinct recombination event between an isotype and A56, a combination that has been relatively uncommon in previous studies on RV recombination. The identification of new recombinant strains in this study provides valuable insights and theoretical evidence regarding the importance of viral recombination in shaping the genetic diversity of RV.

Viral nucleic acid sequence is important original data in the study of viral genetic diversity, genome characteristics, antiviral strategy, and virus tracing. RVs have many types, and molecular classification standards have been revised (for example, the types A44, A95, and A98 have been abolished and combined into types A29, A8, and A54, respectively) (18, 19). There are many RV sequences in GenBank with incomplete genome segment annotations and inaccurate or incomplete type annotations (Fig. 4A). Therefore, we aimed to explore the genetic diversity of RV by systematically mining and annotating all the existing genomic sequences from RVs available in public databases. Through the exploration of RV sequence and the elaboration of genetic diversity of novel types, the data were systematically sorted out and used, and the type information of RV was supplemented. Based on this work, we believe that the unique genetic characteristics of RV support the establishment of an independent RV database resource and platform for data collection and storage, similar to influenza virus (Influenza Research Database) and hepatitis B virus (37, 38), so as to promote the research of RV in a standardized, efficient, and precise way.

In this study, we also investigated the variations in genomic GC content among different serotypes of RV for the first time, utilizing a meticulously annotated and high-quality data set. Our findings revealed significant differences in GC content among various RV serotypes, and these differences were found to be associated with the underlying phylogenetic relationships. Among the selected clades, we observed notable variations in GC content. However, the distribution patterns of GC content within each clade or serotype did not exhibit a clear correlation with the phylogenetic relationships in RVA and RVC. In contrast, within RVB, we identified three distinct clades (b1, b2, and b3) that displayed significant differences in GC content (Fig. 5). Some studies have speculated that the variation in RV GC content may be linked to its adaptation for survival in the upper respiratory tract, specifically in response to temperature conditions (22). Furthermore, certain findings provide support for the hypothesis that RVC, characterized by a higher GC content, exhibits enhanced growth capabilities at core body temperature (23). Based on our findings, additional experiments are warranted in the future to validate whether the observed differences in GC content among serotypes or clades correspond to alterations in optimal survival temperatures or other consequential effects.

By analyzing the evolutionary dynamics of RVA, RVB, and RVC, we revealed the evolutionary relationship, mean evolutionary rate, and effective population size of each type within the species on the time scale. The present study is the first large-scale and systematic analysis of the phylogenetic dynamics of RV. Although RVC was reported late because it could not be cultured *in vitro*, the results showed that RVA had the lowest mean root age, RVC was second, and RVB was the latest RV (Fig. 6A). Among the three RVs, RVB had the lowest mean evolutionary rate of VP1, RVA was slightly higher than RVB, although RVA had the largest number of types (80). The mean evolutionary rate of RVC was the highest, which was due to the high evolution rate of many RVC types, such as C15 and C9. It is speculated that this may be related to the selection pressure and adaptation with the host. Multiple studies similar to ours have reported increasing positive detection rates of RV as RVA, RVC, and RVB (Fig. 1A) (9, 39, 40), the estimated effective population size was approximately consistent with it, but RVA did not produce a significant *N*e(t) advantage compared with RVC after 2000. In the future, further research is necessary to expand the scope of clinical RV detection, enabling the collection of a larger number of sequences.

Together, this study constitutes a comprehensive analysis of the genetic diversity and characterization of RVs in China, which provide new insights on the diversification and emergence of novel RVs types. Some of our analyses and conclusions might be confounded by sample bias and under sampling of RVs. Complete and comprehensive studies on SARS-CoV-2 have shown the power of genomics to our understand of virus pathobiology and transmission (41 – 43). We are hoping that our study provides a foundation for future studies related to the genetic diversity and transmission in China and globally, which will ultimately help on the design of antivirals and vaccines to RVs.

## MATERIALS AND METHODS

### Clinical sample collection and next generation sequencing (NGS)

We collected a total of 52 clinical samples for this study, spanning the period from 2014 to 2018. The samples were obtained from Beijing Children’s Hospital Affiliated with Capital Medical University in Beijing, China, as well as Hebei Children’s Hospital in Hebei Province, China. The extraction of viral nucleic acids from the samples was performed in a BSL-2 facility using an optimized method, which we previously reported (44). Libraries were constructed using the Nextera XT Library Prep Kit (Illumina, San Diego, CA, USA) according to the manufacturer’s instructions. AMPure XP beads were used to clean up the libraries, and the sizes of fragments were assessed using an Agilent 2100 bioanalyzer system (Thermo Fisher). Sequencing was performed on an Illumina HiSeq XTen.

### Genome assembly and annotation

The raw sequencing data were quality controlled using Trimmomatic (V0.32) (45), and the quality-controlled reads were aligned to hg38 using bowtie2 to remove human host contamination (46). Then, *de novo* assembly was performed using Megahit (v1.2.9) to produce contigs, which were compared and annotated using DIAMOND (47). Taxonomic information was viewed by Megan, and RV sequences were obtained (48). The open reading frames and genome segments of the genome sequences were annotated using Geneious Prime 2022.1.1 (https://www.geneious.com). Online BLAST and calculation of the VP1 *p*-distance between sample viruses and prototype strains were used for RV typing.

### Sanger sequencing

For sequences generated by next-generation sequencing, Sanger sequencing was required to fill in the gap and verify the nucleic acid base sequence. Primers were designed by Primer Premier 5 (Premier Biosoft International) for the reference sequence based on the corresponding RV type. The entire reaction volume was 50 µL, including 1 µL of double-stranded deoxyribonucleic acid (dsDNA) as a template, 2 µL of forward primer and reverse primer, 25 µL of Premix Taq (Takara, Japan), and 20-µL nuclease-free water. Thermal cycling conditions were as follows: 94°C 5 min, 94°C 40 cycles for 30 s, 50°C for 30 s, 72°C for 2 min, and then 72°C for 10 min. The PCR products formed by the above operations were sequenced by the Sanger method.

### Identity analysis

After obtaining the viral sequences with relatively complete annotation information and the corresponding typing information of RVs, we calculated the pairwise identities of these sequences with the existing viral sequences of the same type in the GenBank (49). We visualized the data using the ggpubr R package. All multiple sequence alignments in this study were performed with MAFFT (v7.505) (50), and the sequences in the above GenBank database were annotated by Geneious Prime 2022.1.1. We calculated the identity using Python scripts that we developed. In order to maintain the independence of this analysis, we refrained from merging the sequences that we obtained through data mining with the RV types annotated in the Results section during the calculation process.

### Phylogenetic analysis

Before constructing the ML phylogenetic trees in this study, sequence saturation was evaluated using DAMBE software (51, 52). The analysis revealed that all sequences of ML trees exhibited little saturation. Phylogenetic analysis of the complete genome and VP1 genes was performed using the ML method available in MEGA version 11 (53) with 1,000 bootstrap replicates. Complete genome tree employing an optimal nucleotide substitution model of Tamura-Nei distances and a gamma distribution with invariant sites (G + I) and VP1 tree employing the GTR nucleotide substitution model and a gamma distribution of rate variation among sites (G + I) were recommended by the models function in the MEGA version 11. All resulting phylogenetic trees were visualized using iTOL (54).

### Recombination analysis

Multiple sequence alignment was used prior to the detection of recombination events. As described above, the detection of recombination events was performed using RDP (v4.0.1) (27). Because it is difficult to demonstrate the optimal recombination event detection performance under any circumstances relying on a single method, we considered recombination events detected simultaneously by equal to or more than three programs with a median *P*-value of <0.00001 to be reliable. The stand-alone programs included RDP (55), GENECONV (56), Bootscan (57), Maxchi (58), Chimaera (59), SiSscan (60), PhylPro (61), LARD (62), and 3Seq (63). Predicted recombination events were also reassessed by breakpoint polishing, phylogenetic analysis, and alignment consistency checks.

### RNA secondary structure prediction and protein visualization

Based on the primary structure of RNA, its secondary structure could be predicted, which is beneficial to understand the genetic diversity of RV in a new dimension. This study predicts the secondary structure of the UTR of RV using mFOLD (64). The parameters of mFOLD used included a folding temperature of 37°C, an ionic condition of 1 M NaCl with no divalent ions, and a 5% suboptimality. The upper bound of the number of computed folding and the maximum upper bound of the total number of single-stranded bases allowed in a bulge or interior loop were set at 30. The other parameters were set at default, and the initial 1 G was selected as the smallest structure to obtain the Vienna format file. The VARNA (65) software was used for visualization of the RNA secondary structure. The protein visualization was performed using PyMol software.

### Data set construction and VP1 annotation

As of 12 May 2022, we conducted a comprehensive search and downloaded all available GenBank files for RVA, RVB, and RVC using the search term “((Rhinovirus A[Organism]) OR Rhinovirus B[Organism]) OR Rhinovirus C[Organism].” This search yielded 7,921 RVA, 1,687 RVB, and 5,921 RVC nucleic acid GenBank files. Due to incomplete or incorrect sequence annotation in the majority of these files, we meticulously annotated the 15,529 nucleic acid entries using a combination of Geneious Prime 2022.1.1, manual screening, and Python scripts to construct a comprehensive and well-annotated RV data set.

Following the two previous important studies on RV molecular typing (18, 19) and the prototype strain information of the Picornaviridae Study Group Pages (https://www.picornastudygroup.com/), low-quality sequences were filtered out to construct a data set of RV prototype strains (Table S2).

Then, the VP1 *p*-distance between the RV prototype strains and the unannotated strains with the VP1 sequence and the sequence integrity greater than 90% was calculated by Python script. RV typing was then performed according to the molecular typing thresholds of RVA 13%, RVB 12%, and RVC 13%.

Finally, we sorted and summarized the typing results and utilized GraPhlAn to generate high-quality circular representations of the taxonomic tree (66). Subsequently, we utilized the aforementioned iTOL to incorporate statistical information and enhance the visual aesthetics of the taxonomic tree.

### Evolutionary dynamics analysis

Once the RV complete annotation data set was constructed, we performed data mining, checked meta-information, and performed typing to assemble a comprehensive RV VP1 sequence data set. We filtered out VP1 sequences lacking collection date information or with low quality and combined them with the sequences obtained from our samples. This resulted in a data set consisting of 1,078 RVA sequences, 223 RVB sequences, and 687 RVC sequences. These three datasets were utilized for analyzing the evolutionary dynamics.

First, we used the BETS (67, 68) method to test the temporal signal. This method requires a pair of models: a heterochronous model (*M*_het_) and an isochronous model (*M*_iso_), by using a generalized stepping-stone sampling method to obtain the marginal likelihood estimation of the two models and finally applying the Bayes Factor method to determine which model is more suitable for the analyzed data set. A (log) Bayes factor log[*P*(Y|*M*_het_)] − log[*P*(Y|*M*_iso_)] of at least 5 indicated “super strong” support for *M*_het_ over *M*_iso_.

The evolutionary rate and tMRCA were estimated using the BEAST package (v 1.10.5) (69). We selected GTR as the substitution model and gamma + invariant sites as the site heterogeneity model and an uncorrelated lognormal relaxed molecular clock (70). Convergence and mixing were examined using the Tracer program (v1.7.2) (71) with a burn-in period of 10% of the total chain length. In addition, we used a coalescent-based Bayesian Skyline (72) prior for the tree topologies to model the effective population size over time. All parameter estimates yielded effective sampling size >200. The final MCC tree was generated using TreeAnnotator (version 1.10.5) and illustrated in Figtree (version 1.4.4) ( http://tree.bio.ed.ac.uk/software/figtree/).

## Data Availability

The 52 genomic sequences generated in this study have been submitted to the National Center for Biotechnology Information’s GenBank (accession numbers OP342691–OP342742). The raw NGS reads in this study have been deposited in the NCBI Sequence Read Archive (SRA) database under BioProject accession number PRJNA999684 (SRA accession numbers: SRR25448798 -SRR25448849).

## SUPPLEMENTAL MATERIAL

Supplemental material spectrum.00840-23-s0001.pdf Fig. S1 to S5 and Tables S1 to S5.

ASM does not own the copyrights to Supplemental Material that may be linked to, or accessed through, an article. The authors have granted ASM a non-exclusive, world-wide license to publish the Supplemental Material files. Please contact the corresponding author directly for reuse.
