# Supplementary material for: Genetic diversity and characterization of rhinoviruses from Chinese clinical samples with a global perspective
Source: Microbiol Spectr. 2023 Sep 21;11(6):e00840-23. doi: 10.1128/spectrum.00840-23 (PMC10715137; doi:10.1128/spectrum.00840-23)
Supplement: Supplemental material — Fig. S1 to S5 and Tables S1 to S5. [file spectrum.00840-23-s0001.pdf]

Supplementary Tables and Figures

Supplementary Tables

Table1: RV Clinical Sample and Nucleic Acid Sequence Information Table. This table records 52 points of rhinovirus sample information and sequence information, of which the first detection in Asia and the first detection in China are defined by screening the “country”, “collection_date”, and other fields in the Genbank database (as of May 12, 2022).

Case ID	Lab number	Location	Collection date	Species	Type	Strain	Length	First detected in Asia	First detected in China	Novel type	Accession number
1	H6	Hebei	03-Apr-2014	RVA	A56	RVA56/China/2014/HBH6	7109	no	yes	no	OP342721
2	H10	Hebei	12-Apr-2014	RVC	C5	RVC5/China/2014/HBH10	7106	no	no	no	OP342703
3	H13	Hebei	22-Apr-2014	RVC	C42	RVC42/China/2014/HBH13	7112	no	yes	no	OP342691
4	H14	Hebei	25-Apr-2014	RVA	A15	RVA15/China/2014/HBH14	7122	no	yes	no	OP342737
5	H17	Hebei	07-May-2014	RVA	A68	RVA68/China/2014/HBH17	7101	no	yes	no	OP342715
6	H18	Hebei	28-Apr-2014	RVA	A106	RVA106/China/2014/HBH18	7153	yes	yes	no	OP342709
7	H20	Hebei	29-Apr-2014	RVA	A56	RVA56/China/2014/HBH20	7123	no	yes	no	OP342720
8	H24	Hebei	06-May-2014	RVA	A58	RVA58/China/2014/HBH24	7170	no	yes	no	OP342718
9	H29	Hebei	16-May-2014	RVA	A9	RVA9/China/2014/HBH29	7132	no	yes	no	OP342742
10	H30	Hebei	19-May-2014	RVC	C15	RVC15/China/2014/HBH30	7132	no	no	no	OP342700
11	H33	Hebei	10-Jun-2014	RVA	A56	RVA56/China/2014/HBH33	7042	no	yes	no	OP342719
12	H41	Hebei	27-Oct-2014	RVC	C22	RVC22/China/2014/HBH41	7090	no	yes	no	OP342696
13	H43	Hebei	01-Nov-2014	RVA	A12	RVA12/China/2014/HBH43	7109	no	no	no	OP342740
14	H55	Hebei	07-Feb-2015	RVA	A34	RVA34/China/2015/HBH55	7147	no	no	no	OP342731
15	H67	Hebei	07-Oct-2014	RVA	A49	RVA49/China/2014/HBH67	7149	no	no	no	OP342726
16	H68	Hebei	18-May-2015	RVA	A80	RVA80/China/2015/HBH68	7139	no	yes	no	OP342713
17	H70	Hebei	17-Jun-2015	RVA	A12	RVA12/China/2015/HBH70	7161	no	no	no	OP342739
18	H73	Hebei	02-Jul-2015	RVB	B3	RVB3/China/2015/HBH73	7232	no	yes	no	OP342707
19	H77	Hebei	09-Jul-2015	RVA	A10	RVA10/China/2015/HBH77	7138	no	yes	no	OP342741
20	P4	Beijing	24-Feb-2017	RVA	A28	RVA28/China/2017/BJP4	7143	no	yes	no	OP342734
21	P45	Beijing	12-May-2017	RVA	A38	RVA38/China/2017/BJP45	7137	no	no	no	OP342729
22	P66	Beijing	14-Jun-2017	RVA	A12	RVA12/China/2017/BJP66	7099	no	no	no	OP342738
23	P21	Beijing	30-Mar-2017	RVA	A22	RVA22/China/2017/BJP21	7070	no	no	no	OP342736
24	P37	Beijing	09-May-2017	RVA	A103	RVA103/China/2017/BJP37	7062	no	yes	no	OP342710
25	P14	Beijing	13-Mar-2017	RVA	A110	RVA110/China/2017/BJP14	7097	yes	yes	yes	OP342708
26	P59	Beijing	31-May-2017	RVC	C24	RVC24/China/2017/BJP59	7056	no	yes	no	OP342695
27	P7	Beijing	03-Mar-2017	RVC	C6	RVC6/China/2017/BJP7	7028	no	no	no	OP342702
28	P1	Beijing	09-Feb-2017	RVC	C15	RVC15/China/2017/BJP1	6895	no	no	no	OP342699
29	P22	Beijing	31-Mar-2017	RVA	A22	RVA22/China/2017/BJP22	6968	no	no	no	OP342735
30	P31	Beijing	10-Apr-2017	RVA	A80	RVA80/China/2017/BJP31	6846	no	yes	no	OP342712
31	R5	Beijing	29-Jan-2017	RVC	C26	RVC26/China/2017/BJR5	7122	no	yes	no	OP342694
32	R7	Beijing	29-Jan-2017	RVA	A54	RVA54/China/2017/BJR7	7129	no	no	no	OP342723
33	R11	Beijing	30-Jan-2018	RVC	C35	RVC35/China/2018/BJR11	7104	no	yes	no	OP342693
34	R25	Beijing	30-Jan-2018	RVA	A76	RVA76/China/2018/BJR25	7129	yes	yes	no	OP342714

35	R26	Beijing	30-Jan-2018	RVA	A53	RVA53/China/2018/BJR26	7123	no	no	no	OP342724
36	R28	Beijing	26-Dec-2017	RVC	C36	RVC36/China/2017/BJR28	7057	no	yes	no	OP342692
37	R32	Beijing	26-Dec-2017	RVC	C15	RVC15/China/2017/BJR32	7205	no	no	no	OP342698
38	R37	Beijing	31-Jan-2018	RVA	A54	RVA54/China/2018/BJR37	7161	no	no	no	OP342722
39	R42	Beijing	31-Jan-2018	RVB	B3	RVB3/China/2018/BJR42	6991	no	yes	no	OP342706
40	R46	Beijing	01-Feb-2018	RVC	C13	RVC13/China/2018/BJR46	7046	no	yes	no	OP342701
41	R48	Beijing	26-Dec-2017	RVA	A33	RVA33/China/2017/BJR48	7144	no	no	no	OP342732
42	R50	Beijing	02-Feb-2018	RVB	B3	RVB3/China/2018/BJR50	6940	no	yes	no	OP342705
43	R59	Beijing	28-Dec-2017	RVA	A30	RVA30/China/2017/BJR59	7126	no	no	no	OP342733
44	R66	Beijing	05-Feb-2018	RVB	B3	RVB3/China/2018/BJR66	7145	no	yes	no	OP342704
45	R68	Beijing	05-Feb-2018	RVA	A60	RVA60/China/2018/BJR68	7077	no	yes	no	OP342717
46	R73	Beijing	06-Feb-2018	RVC	C15	RVC15/China/2018/BJR73	7049	no	no	no	OP342697
47	R83	Beijing	06-Feb-2018	RVA	A34	RVA34/China/2018/BJR83	6996	no	no	no	OP342730
48	R100	Beijing	08-Feb-2018	RVA	A89	RVA89/China/2018/BJR100	7126	no	no	no	OP342711
49	R108	Beijing	09-Feb-2018	RVA	A45	RVA45/China/2018/BJR108	7108	no	no	no	OP342728
50	R109	Beijing	09-Feb-2018	RVA	A51	RVA51/China/2018/BJR109	7156	no	yes	no	OP342725
51	R122	Beijing	12-Feb-2018	RVA	A45	RVA45/China/2018/BJR122	7149	no	no	no	OP342727
52	R129	Beijing	13-Feb-2018	RVA	A67	RVA67/China/2018/BJR129	7156	yes	yes	no	OP342716

Table2: RV Prototype Strain Information Table. These are all the prototype rhinovirus strains used in this study. The "Genome" and "VP1" fields represent the presence or absence of the complete genome and VP1 sequence, respectively.

Specie	Number	Type	Accession Number	Length(nt)	Date of submission	Country	Genome	VP1
RVA	1	A1	D00239	7133	2020-10-03		yes	yes
RVA	2	A2	X02316	7102	2006-11-14		yes	yes
RVA	3	A7	DQ473503	7146	2022-01-24	USA	yes	yes
RVA	4	A8	FJ445113	7108	2009-04-10		yes	yes
RVA	5	A9	FJ445177	7132	2009-04-10		yes	yes
RVA	6	A10	DQ473498	7137	2022-01-24	USA	yes	yes
RVA	7	A11	EF173414	7125	2007-07-15		yes	yes
RVA	8	A12	EF173415	7124	2007-07-15		yes	yes
RVA	9	A13	FJ445116	7140	2009-04-10		yes	yes
RVA	10	A15	DQ473493	7134	2022-01-24	USA	yes	yes
RVA	11	A16	L24917	7124	1995-07-14		yes	yes
RVA	12	A18	FJ445118	7119	2009-04-10		yes	yes
RVA	13	A19	FJ445119	7135	2009-04-10		yes	yes
RVA	14	A20	FJ445120	7163	2009-04-10		yes	yes
RVA	15	A21	FJ445121	7134	2009-04-10		yes	yes
RVA	16	A22	FJ445122	7129	2009-04-10		yes	yes
RVA	17	A23	DQ473497	7025	2022-01-24	USA	yes	yes
RVA	18	A24	EF173416	7132	2007-07-15		yes	yes
RVA	19	A25	FJ445123	7126	2009-04-10		yes	yes
RVA	20	A28	DQ473508	7148	2022-01-24	USA	yes	yes

RVA	21	A29	DQ473499	7123	2022-01-24	USA	yes	yes
RVA	22	A30	DQ473512	7099	2022-01-24	USA	yes	yes
RVA	23	A31	FJ445126	7131	2009-04-10		yes	yes
RVA	24	A32	FJ445127	7133	2009-04-10		yes	yes
RVA	25	A33	FJ445128	7133	2009-04-10		yes	yes
RVA	26	A34	DQ473501	7119	2022-01-24	USA	yes	yes
RVA	27	A36	DQ473505	7141	2022-01-24	USA	yes	yes
RVA	28	A38	DQ473495	7136	2022-01-24	USA	yes	yes
RVA	29	A39	AY751783	7137	2005-04-12	USA	yes	yes
RVA	30	A40	FJ445129	7138	2009-04-10		yes	yes
RVA	31	A41	DQ473491	7145	2022-01-24	USA	yes	yes
RVA	32	A43	FJ445131	7129	2009-04-10		yes	yes
RVA	33	A45	FJ445132	7114	2009-04-10		yes	yes
RVA	34	A46	DQ473506	7149	2022-01-24	USA	yes	yes
RVA	35	A47	FJ445133	7132	2009-04-10		yes	yes
RVA	36	A49	DQ473496	7106	2022-01-24	USA	yes	yes
RVA	37	A50	FJ445135	7118	2009-04-10		yes	yes
RVA	38	A51	FJ445136	7152	2009-04-10		yes	yes
RVA	39	A53	DQ473507	7143	2022-01-24	USA	yes	yes
RVA	40	A54	FJ445138	7134	2009-04-10		yes	yes
RVA	41	A55	DQ473511	7036	2022-01-24	USA	yes	yes
RVA	42	A56	FJ445140	7136	2009-04-10		yes	yes
RVA	43	A57	FJ445141	7134	2009-04-10	USA	yes	yes
RVA	44	A58	FJ445142	7140	2009-04-10		yes	yes
RVA	45	A59	DQ473500	7135	2022-01-24	USA	yes	yes
RVA	46	A60	FJ445143	7139	2009-04-10		yes	yes
RVA	47	A61	FJ445144	7139	2009-04-10		yes	yes
RVA	48	A62	FJ445145	7131	2009-04-10		yes	yes
RVA	49	A63	FJ445146	7141	2009-04-10		yes	yes
RVA	50	A64	EF173417	7129	2007-07-15		yes	yes
RVA	51	A65	FJ445147	7162	2009-04-10		yes	yes
RVA	52	A66	FJ445148	7139	2009-04-10		yes	yes
RVA	53	A67	FJ445149	7135	2009-04-10		yes	yes
RVA	54	A68	FJ445150	7164	2009-04-10		yes	yes
RVA	55	A71	FJ445152	7161	2009-04-10		yes	yes
RVA	56	A73	DQ473492	7140	2022-01-24	USA	yes	yes
RVA	57	A74	DQ473494	7120	2022-01-24	USA	yes	yes
RVA	58	A75	DQ473510	7137	2022-01-24	USA	yes	yes
RVA	59	A76	DQ473502	7129	2022-01-24	USA	yes	yes
RVA	60	A77	FJ445154	7136	2009-04-10		yes	yes
RVA	61	A78	EF173418	7145	2007-07-15		yes	yes
RVA	62	A80	FJ445156	7138	2009-04-10		yes	yes
RVA	63	A81	FJ445157	7116	2009-04-10		yes	yes
RVA	64	A82	DQ473509	7129	2022-01-24	USA	yes	yes

RVA	65	A85	FJ445163	7140	2009-04-10		yes	yes
RVA	66	A88	DQ473504	7143	2022-01-24	USA	yes	yes
RVA	67	A89	M16248	7152	2003-02-07		yes	yes
RVA	68	A90	FJ445167	7124	2009-04-10		yes	yes
RVA	69	A94	EF173419	7132	2007-07-15		yes	yes
RVA	70	A96	FJ445171	7134	2009-04-10		yes	yes
RVA	71	A100	FJ445175	7140	2009-04-10		yes	yes
RVA	72	A101	GQ415051	7130	2022-02-22	USA	yes	yes
RVA	73	A102	EF155421	7160	2010-07-31	Netherlands	yes	yes
RVA	74	A103	JF965515	7168	2011-06-02	Australia	yes	yes
RVA	75	A104	JN562727	6843	2017-01-25	USA	yes	yes
RVA	76	A105	JN614995	6709	2017-01-25	USA	yes	yes
RVA	77	A106	JX025555	6728	2017-01-25	USA	yes	yes
RVA	78	A107	KC859319	990	2020-10-16	Spain	no	yes
RVA	79	A108	KC859318	1008	2020-10-16	Spain	no	yes
RVA	80	A109	KP737114	1355	2017-01-19	Germany	no	yes
RVB	1	B3	DQ473485	7208	2022-01-24	USA	yes	yes
RVB	2	B4	DQ473490	7212	2022-01-24	USA	yes	yes
RVB	3	B5	FJ445112	7212	2009-04-10		yes	yes
RVB	4	B6	DQ473486	7216	2022-01-24	USA	yes	yes
RVB	5	B14	L05355	7212	1993-06-11		yes	yes
RVB	6	B17	EF173420	7219	2007-07-15		yes	yes
RVB	7	B26	FJ445124	7211	2009-04-10		yes	yes
RVB	8	B27	EF173421	7217	2007-07-15		yes	yes
RVB	9	B35	DQ473487	7224	2022-01-24	USA	yes	yes
RVB	10	B37	EF173423	7216	2007-07-15		yes	yes
RVB	11	B42	FJ445130	7223	2009-04-10		yes	yes
RVB	12	B48	DQ473488	7214	2022-01-24	USA	yes	yes
RVB	13	B52	EF173424	7216	2007-07-15		yes	yes
RVB	14	B69	FJ445151	7211	2009-04-10		yes	yes
RVB	15	B70	DQ473489	7223	2022-01-24	USA	yes	yes
RVB	16	B72	FJ445153	7216	2009-04-10		yes	yes
RVB	17	B79	FJ445155	7224	2009-04-10		yes	yes
RVB	18	B83	FJ445161	7230	2009-04-10		yes	yes
RVB	19	B84	FJ445162	7201	2009-04-10		yes	yes
RVB	20	B86	FJ445164	7213	2009-04-10		yes	yes
RVB	21	B91	FJ445168	7221	2009-04-10		yes	yes
RVB	22	B92	FJ445169	7233	2009-04-10		yes	yes
RVB	23	B93	EF173425	7215	2007-07-15		yes	yes
RVB	24	B97	FJ445172	7207	2009-04-10		yes	yes
RVB	25	B99	FJ445174	7208	2009-04-10		yes	yes
RVB	26	B100	HQ123444	6537	2020-10-16	Thailand	yes	yes
RVB	27	B101	JF781500	6920	2020-10-16	USA	yes	yes
RVB	28	B102	JX074053	7189	2020-10-16	USA	yes	yes

RVB	29	B103	JN614996	6899	2020-10-16	USA	yes	yes
RVB	30	B104	FJ445137	7216	2009-04-10	USA	yes	yes
RVB	31	B105	KP736666	1370	2017-01-19	Belgium	no	yes
RVB	32	B106	KP736636	1433	2017-01-19	Spain	no	yes
RVC	1	C1	EF077279	6944	2022-02-01		yes	yes
RVC	2	C2	EF077280	7015	2022-02-01		yes	yes
RVC	3	C3	EF186077	7134	2013-01-23	Australia	yes	yes
RVC	4	C4	EF582385	7099	2022-02-01	China	yes	yes
RVC	5	C5	EF582386	7114	2022-02-01	China	yes	yes
RVC	6	C6	EF582387	7086	2022-02-01	China	yes	yes
RVC	7	C7	DQ875932	7072	2022-02-01	USA	yes	yes
RVC	8	C8	GQ223227	7107	2022-02-22	China	yes	yes
RVC	9	C9	GQ223228	7111	2022-02-22	China	yes	yes
RVC	10	C10	GQ323774	7010	2010-03-24	Australia	yes	yes
RVC	11	C11	EU840952	7108	2012-10-05	Switzerland	yes	yes
RVC	12	C12	HM236958	834	2022-02-25	United Kingdom	no	yes
RVC	13	C13	HM236908	819	2022-02-25	United Kingdom	no	yes
RVC	14	C14	HM236911	822	2022-02-25	United Kingdom	no	yes
RVC	15	C15	HM236963	840	2022-02-25	United Kingdom	no	yes
RVC	16	C16	HM236944	822	2022-02-25	United Kingdom	no	yes
RVC	17	C17	HM236936	822	2022-02-25	United Kingdom	no	yes
RVC	18	C18	HM236918	840	2022-02-25	United Kingdom	no	yes
RVC	19	C19	EU840728	3055	2022-02-01	Switzerland	no	yes
RVC	20	C20	HM236923	828	2022-02-25	United Kingdom	no	yes
RVC	21	C21	HM236903	822	2022-02-25	United Kingdom	no	yes
RVC	22	C22	HM236905	822	2022-02-25	United Kingdom	no	yes
RVC	23	C23	HM236901	822	2022-02-25	United Kingdom	no	yes
RVC	24	C24	HM236939	822	2022-02-25	United Kingdom	no	yes
RVC	25	C25	HM236952	809	2022-02-25	United Kingdom	no	yes
RVC	26	C26	HM236904	822	2022-02-25	United Kingdom	no	yes
RVC	27	C27	HM236906	813	2022-02-25	United Kingdom	no	yes
RVC	28	C28	HM236954	831	2022-02-25	United Kingdom	no	yes
RVC	29	C29	HM236949	822	2022-02-25	United Kingdom	no	yes
RVC	30	C30	HM236968	816	2022-02-25	United Kingdom	no	yes
RVC	31	C31	HM236964	797	2022-02-25	United Kingdom	no	yes
RVC	32	C32	HM236897	816	2022-02-25	United Kingdom	no	yes
RVC	33	C33	HM236934	834	2022-02-25	United Kingdom	no	yes
RVC	34	C34	JF519758	852	2020-10-16	Papua New Guinea	no	yes
RVC	35	C35	JF436925	6864	2013-11-21	Sweden	yes	yes
RVC	36	C36	JF416311	810	2020-10-16	United Kingdom	no	yes
RVC	37	C37	JF416321	812	2020-10-16	United Kingdom	no	yes
RVC	38	C38	JF416322	815	2020-10-16	United Kingdom	no	yes
RVC	39	C39	JN205461	7115	2012-07-01	Australia	yes	yes

RVC	40	C40	JN815251	6613	2017-01-25	USA	yes	yes
RVC	41	C41	KF958311	7115	2015-11-17	USA	yes	yes
RVC	42	C42	JF416320	818	2020-10-16	United Kingdom	no	yes
RVC	43	C43	KY369878	7052	2017-04-19	USA	yes	yes
RVC	44	C44	JF416310	833	2020-10-16	United Kingdom	no	yes
RVC	45	C45	JF416308	815	2020-10-16	United Kingdom	no	yes
RVC	46	C46	JF416318	815	2020-10-16	United Kingdom	no	yes
RVC	47	C47	JF519760	837	2020-10-16	Papua New Guinea	no	yes
RVC	48	C48	JF519762	840	2020-10-16	Papua New Guinea	no	yes
RVC	49	C49	JF946738	853	2016-07-25	Australia	no	yes
RVC	50	C50	KF688606	6928	2020-10-16	Australia	yes	yes
RVC	51	C51	JF317015	7154	2020-10-16	China	yes	yes
RVC	52	C52	KP736724	1287	2017-01-19	Spain	no	yes
RVC	53	C53	MK989756	7151	2020-09-22	Kenya	yes	yes
RVC	54	C54	KP282614	7054	2015-02-02	Spain	yes	yes
RVC	55	C55	KR997885	3124	2015-12-17	Tanzania	no	yes
RVC	56	C56	LC004772	2343	2015-02-28	Japan	no	yes
RVC	57	C57	KP890662	7073	2016-05-24	Malaysia	yes	yes

Table3: Recombination events. The nine right-most columns in the table represent the *P*-values of the nine recombination assays in RDP.

Number	Recombinant Sequence(s)	Minor Parental Sequence(s)	Major Parental Sequence(s)	Start	End	*P*-RDP	*P*-GENECONV	*P*-Bootscan	*P*-Maxchi	*P*-Chimaera	*P*-SiSscan	*P*-PhylPro	*P*-LARD	*P*-3Seq
1	**RVA67/China/2018/BJR129**<br>OK181491 RvA67/USA/2021/PFJBXD 2021 A67<br>MZ322931 RvA67/USA/2021/YAEPRB 2021 A67	DQ473505 A36	FJ445149 ATCC_VR-1177 A67	6	572	1.07E-24	9.13E-42	1.63E-20	1.09E-05	1.02E-05	1.94E-06	NS	NS	5.71E-10
2	**RVA76/China/2018/BJR25**	DQ473502 A76<br>FJ445182 ATCC_VR-1186 A76	EU840727 A56<br>FJ445140 ATCC_VR-1166 A56	28	5196	1.25E-05	1.23E-12	1.78E-07	6.79E-13	1.64E-11	4.50E-32	NS	NS	5.29E-25
3	**RVA80/China/2015/HBH68**<br>**RVA80/China/2017/BJP31**<br>JN798576 HRV-A80_p1183_sR1526_2009 A80<br>JN990705 HRV-A80_p1187_sR2954_2009 2009 A80<br>OK181467 RvA80/USA/2021/AE4MDS 2021 A80<br>MZ835564 RvA80/USA/2021/8CHZMC 2021 A80<br>FJ445156 ATCC_VR-1190 A80	DQ473499.1 A29	FJ445147 ATCC_VR-1175 A65<br>EF155421 AMS323 A102	12	758	8.52E-15	NS	5.52E-10	6.86E-04	1.23E-05	5.07E-08	NS	NS	5.71E-10
4	**RVA38/China/2017/BJP45**<br>MW587063 S0614ZSZ 2016 A38<br>JQ994496 HRV-A38_p1266_s3799_1999 1999 A38<br>DQ473495 A38<br>FJ445180 ATCC_VR-1148 A38	**RVA15/China/2014/HBH14**<br>MW713789 7061 A15	FJ445138 ATCC_VR-1164 A54<br>**RVA54/China/2017/BJR7**<br>**RVA54/China/2018/BJR37**<br>MW587081 S0858HXJ 2016 A54<br>LC699416 Fukushima_H504_2019 2019 A54<br>MW587090 S0939LX 2016 A54<br>OK539505 RvA54/USA/2021/SLPWR2 2021 A54	1049	5196	NS	NS	NS	1.50E-07	1.18E-04	5.91E-07	NS	NS	2.86E-10
5	**RVC5/China/2014/HBH10**<br>EF582386 25 C5<br>KY369880 SC261 2016 C5<br>EU840952 CL-170085 C11	MF806525 CA-RGDS-1001 C47	KP282614 D3490 C54	33	673	1.59E-09	3.91E-07	6.24E-08	1.35E-02	0.011931829	1.37E-07	NS	NS	6.77E-05
6	HQ123444 CU211 B100	DQ473487 B35	JX074053 HRV-B92_p1044_sR122_2007 2007 B102	4514	7022	2.04E-17	9.05E-26	2.92E-09	0.023997834	5.89E-06	NS	NS	NS	NS
7	AY751783 A39	FJ445178 ATCC_VR-1120 A10<br>**RVA10/China/2015/HBH77**<br>DQ473498 A10	DQ473512 A30<br>**RVA30/China/2017/BJR59**<br>MW587051 S0135LYF 2016 A30<br>MH426976 MCL-18-H-1135 2018 A30	649	3152	NS	NS	NS	1.88E-04	6.23E-06	0.021013452	NS	NS	2.46E-06

Table4: Data mining, cleaning, and RVA type-annotated table. “VP1 *p*-distance” indicates pairwise nucleotide *p*-distance between annotated VP1 sequence and prototype strain VP1. For the new rhinovirus, the table lists the lowest *p*-distance (the comparator prototype strain is noted later). “VP1 Start, End, Length” containing “>” or “<” represents an indeterminate position or truncated end.

Number	RV	Accession	Annotated Types	VP1 Start	VP1 End	VP1 Length	VP1 *p*-distance	The typing threshold(RVA:0.13,RVB:0.12,RVC:0.13) is exceeded
1	A	MW587069	A1	2346	3206	861	0.12659698	
2	A	JQ837724	A1	2285	3145	861	0.131539611	yes, but *p*-distance is located near the threshold of typing and there is no significant phylogenetic difference, so it is not considered as a novel type
3	A	JN837694	A1	2159	3019	861	0.128919861	
4	A	JN815255	A1	2253	3113	861	0.090592334	
5	A	JN798558	A1	2107	2967	861	0.103550296	
6	A	MN488768	A2	1	>791	>791	0.094816688	
7	A	MW587074	A7	2340	3212	873	0.10882016	
8	A	MW587071	A7	2340	3212	873	0.109965636	
9	A	MW587067	A7	2337	3209	873	0.107674685	
10	A	MW587072	A7	2304	3176	873	0.115693013	
11	A	MW587064	A7	1489	2361	873	0.111111111	
12	A	JF285320	A9	2212	3078	867	0.107266436	
13	A	JF285319	A9	2212	3078	867	0.107266436	
14	A	JF285318	A9	2212	3078	867	0.106113033	
15	A	JF285317	A9	2212	3078	867	0.106113033	
16	A	JF285316	A9	2212	3078	867	0.111880046	
17	A	JF285315	A9	2212	3078	867	0.111880046	
18	A	JF285314	A9	2212	3078	867	0.113033449	
19	A	JF285313	A9	2212	3078	867	0.111880046	
20	A	JF285312	A9	2212	3078	867	0.114186851	
21	A	JF285311	A9	2212	3078	867	0.111880046	
22	A	JF285310	A9	2212	3078	867	0.113033449	
23	A	JN541269	A10	2400	3263	864	0.099871959	
24	A	JN798575	A10	2291	3154	864	0.09375	
25	A	JN798582	A10	2236	3099	864	0.094907407	
26	A	JN815247	A10	2216	3079	864	0.096774194	
27	A	LC428146	A10	1	865	865	0.104166667	
28	A	LC428156	A10	1	864	864	0.103009259	
29	A	MK989737	A12	2291	3151	861	0.095238095	
30	A	HQ123441	A12	1711	2571	861	0.066202091	
31	A	JF781511	A12	1765	2625	861	0.098722416	
32	A	MH685689	A12	1921	2781	861	0.099883856	

33	A	LC428145	A12	1	861	861	0.104529617
34	A	MW587066	A13	2253	3122	870	0.082758621
35	A	JN541268	A15	2243	3112	870	0.102298851
36	A	JN562722	A16	2323	3177	855	0.088888889
37	A	MH685687	A16	2318	3172	855	0.095906433
38	A	JN990704	A16	2219	3073	855	0.087719298
39	A	JN798574	A16	2252	3133	882	0.083036773
40	A	JX074057	A16	2234	3088	855	0.085380117
41	A	JN815253	A16	2253	3107	855	0.083235639
42	A	JN614992	A16	2193	3047	855	0.083040936
43	A	LC428152	A16	1	855	855	0.085380117
44	A	LC428140	A16	1	855	855	0.088888889
45	A	JF781496	A18	2247	3107	861	0.082462253
46	A	JF781508	A18	2151	3011	861	0.084785134
47	A	LC428147	A18	1	861	861	0.089430894
48	A	LC428144	A18	1	861	861	0.092915215
49	A	MW587073	A19	2200	3069	870	0.106896552
50	A	JQ747746	A19	2224	3093	870	0.091954023
51	A	JQ747750	A19	2402	3271	870	0.096551724
52	A	LC428143	A19	1	870	870	0.1
53	A	JN614993	A20	2240	3130	891	0.099887767
54	A	JN541270	A20	2151	3041	891	0.092031425
55	A	JN798571	A20	2056	2946	891	0.099887767
56	A	JQ994494	A20	2179	3069	891	0.098765432
57	A	KR871677	A21	2325	3185	861	0.105691057
58	A	KR871676	A21	2325	3185	861	0.095238095
59	A	KR871675	A21	2325	3185	861	0.096399535
60	A	KR871674	A21	2325	3185	861	0.1242741
61	A	MF043119	A21	2325	3185	861	0.095238095
62	A	JN837693	A21	2254	3114	861	0.094076655
63	A	MK501739	A21	2212	3072	861	0.101045296
64	A	JQ747747	A21	2149	3009	861	0.077816492
65	A	MH685688	A21	2299	3159	861	0.092915215
66	A	LC428157	A21	1	861	861	0.092915215
67	A	MW587085	A22	2317	3183	867	0.104959631
68	A	MW587095	A22	2316	3182	867	0.102652826
69	A	MH685692	A22	2308	3174	867	0.092272203
70	A	MH685690	A22	2222	3088	867	0.095732411
71	A	JN837696	A23	2253	3101	849	0.104829211
72	A	JN621244	A23	2270	3118	849	0.103651355
73	A	JN815254	A23	2233	3081	849	0.104829211
74	A	MN488766	A23	1	849	849	0.106007067
75	A	JF285328	A24	2314	3168	855	0.086549708
76	A	JF285327	A24	2314	3168	855	0.086549708

77	A	JF285326	A24	2314	3168	855	0.087719298
78	A	JF285325	A24	2314	3168	855	0.09005848
79	A	JN798563	A24	2259	3113	855	0.077192982
80	A	LC428137	A24	1	855	855	0.09005848
81	A	LC428135	A24	1	855	855	0.09005848
82	A	JQ747751	A28	2200	3078	879	0.10882016
83	A	MK501733	A28	2186	3064	879	0.119129439
84	A	JN798577	A28	1976	2848	873	0.086253369
85	A	JN798580	A28	1941	2813	873	0.084765178
86	A	LC428161	A28	1	873	873	0.087056128
87	A	JN815252	A29	2372	3214	843	0.109134045
88	A	MW587051	A30	2312	3160	849	0.104829211
89	A	MW587094	A30	2225	3073	849	0.10836278
90	A	JN798557	A30	2242	3090	849	0.097777778
91	A	MW587056	A31	2336	3190	855	0.123976608
92	A	MW587057	A33	2324	3181	858	0.086247086
93	A	MW587065	A33	2238	3095	858	0.094405594
94	A	JN990707	A33	2109	2966	858	0.101398601
95	A	JN815250	A33	1873	2730	858	0.101398601
96	A	MN488771	A33	1	858	858	0.099067599
97	A	MW587093	A34	2438	3298	861	0.113821138
98	A	MW587075	A34	2227	3087	861	0.117305459
99	A	MW587070	A34	2314	3174	861	0.114982578
100	A	MW587084	A34	2313	3173	861	0.120789779
101	A	MW587082	A34	2227	3087	861	0.117305459
102	A	JN562720	A34	1896	2756	861	0.096399535
103	A	MW587062	A34	1640	2500	861	0.116144019
104	A	LC428154	A34	1	861	861	0.092915215
105	A	KF958307	A36	2339	3205	867	0.078431373
106	A	JN798583	A36	2267	3133	867	0.076124567
107	A	JF781497	A36	2253	3119	867	0.078431373
108	A	JN837697	A36	2263	3129	867	0.078431373
109	A	JX074050	A36	<2287	3126	>840	0.077380952
110	A	JN614994	A36	2269	3135	867	0.079584775
111	A	JN815246	A36	2269	3135	867	0.07266436
112	A	JN798584	A36	2252	3118	867	0.074971165
113	A	JN815242	A36	2266	3132	867	0.076124567
114	A	JN815241	A36	2253	3119	867	0.076124567
115	A	JN621243	A36	2253	3122	870	0.077011494
116	A	MW587063	A38	2217	3086	870	0.104597701
117	A	JQ994496	A38	2405	3274	870	0.093103448
118	A	JN541272	A38	2318	3187	870	0.100346021
119	A	MW679010	A39	2251	3105	855	0.09005848
120	A	MN488775	A39	1	855	855	0.09122807

121	A	MN488774	A39	1	855	855	0.09005848
122	A	JX074051	A40	2177	3034	858	0.086247086
123	A	JN798579	A40	2247	3104	858	0.085081585
124	A	JQ245967	A40	1938	2795	858	0.086247086
125	A	LC428158	A40	1	858	858	0.08974359
126	A	JN815237	A43	2292	3158	867	0.108419839
127	A	MK989739	A45	2287	3135	849	0.090694935
128	A	LC428163	A45	1	849	849	0.088339223
129	A	MW679008	A46	2295	3173	879	0.068259386
130	A	MK989738	A46	2237	3115	879	0.069397042
131	A	LC428141	A46	1	879	879	0.003412969
132	A	GQ223229	A47	2329	3186	858	0.104093567
133	A	JN837692	A47	2280	3137	858	0.101754386
134	A	MN488767	A47	1	858	858	0.120467836
135	A	MN749155	A49	2313	3160	848	0.095518868
136	A	JN621241	A49	2270	3118	849	0.094228504
137	A	MW587079	A49	2248	3096	849	0.096584217
138	A	KY967355	A49	2259	3106	848	0.099056604
139	A	MW587089	A49	2226	3074	849	0.100117786
140	A	JN798589	A49	2242	3090	849	0.097762073
141	A	LC428153	A49	1	849	849	0.101295642
142	A	JN562725	A51	2017	2904	888	0.093468468
143	A	MW587061	A53	2448	3323	876	0.084474886
144	A	JN798587	A53	2269	3144	876	0.094748858
145	A	MW587081	A54	2448	3305	858	0.097902098
146	A	MW587090	A54	2240	3097	858	0.096736597
147	A	MH685691	A54	2294	3151	858	0.096736597
148	A	MH685686	A54	2295	3152	858	0.096736597
149	A	MH685693	A54	2274	3131	858	0.097902098
150	A	MH685683	A54	685	1542	858	0.090909091
151	A	LC428155	A54	1	858	858	0.121212121
152	A	JQ837718	A55	2260	3120	861	0.054587689
153	A	JX025558	A58	2252	3121	870	0.08045977
154	A	MN488758	A58	1	870	870	0.086206897
155	A	LC428151	A58	1	870	870	0.091954023
156	A	LC428150	A58	1	870	870	0.085057471
157	A	LC428148	A58	1	870	870	0.091954023
158	A	MK989736	A59	2246	3103	858	0.096736597
159	A	MK989740	A59	2241	3098	858	0.088578089
160	A	JN541266	A59	2287	3144	858	0.086247086
161	A	MF118144	A59	2287	3144	858	0.097902098
162	A	MF118143	A59	2288	3145	858	0.095571096
163	A	MK989733	A59	2295	3152	858	0.090909091
164	A	MN488773	A59	1	858	858	0.093240093

165	A	LC428160	A59	1	858	858	0.095571096
166	A	MH899591	A60	2199	3068	870	0.114942529
167	A	JN798590	A60	2112	2981	870	0.096511628
168	A	JN798560	A61	2250	3125	876	0.100472813
169	A	MN488772	A61	1	876	876	0.095890411
170	A	JF285324	A64	2295	3161	867	0.064590542
171	A	JF285323	A64	2295	3161	867	0.06805075
172	A	JF285322	A64	2295	3161	867	0.071510957
173	A	JF285321	A64	2295	3161	867	0.070357555
174	A	MK989734	A65	2313	3200	888	0.081081081
175	A	JF781504	A65	2308	3195	888	0.065315315
176	A	JQ245966	A65	2152	3039	888	0.064189189
177	A	JQ837715	A66	2181	3044	864	0.083333333
178	A	JN621246	A66	1940	2803	864	0.081018519
179	A	JN621245	A67	2256	3122	867	0.094579008
180	A	KT751301	A67	2161	3027	867	0.0911188
181	A	KT751300	A67	2164	3019	856	0.09228972
182	A	MN488770	A67	1	867	867	0.088811995
183	A	JN798578	A68	2263	3153	891	0.102132435
184	A	MN488751	A68	1	891	891	0.11335578
185	A	JN837690	A75	2249	3115	867	0.081845238
186	A	JF781503	A75	2188	3054	867	0.073474471
187	A	LC428162	A75	1	867	867	0.085351788
188	A	JN815238	A76	2029	2889	861	0.089058524
189	A	JX074049	A76	2378	3235	858	0.090909091
190	A	JX074055	A76	1922	2779	858	0.093333333
191	A	MN488765	A76	1	>806	>806	0.083126551
192	A	JN990705	A80	2311	3186	876	0.077625571
193	A	JN798576	A80	2193	3068	876	0.083333333
194	A	JN798586	A80	2121	2996	876	0.074780059
195	A	MH828516	A80	1708	2583	876	0.082191781
196	A	MN488752	A80	1	876	876	0.086757991
197	A	MN488748	A80	1	>864	>864	0.085648148
198	A	HQ123442	A81	1705	2559	855	0.129824561
199	A	LC428159	A81	1	855	855	0.12748538
200	A	JN798556	A82	<2346	3179	>834	0.090078329
201	A	MN306020	A82	2157	3023	867	0.040369089
202	A	JQ837722	A82	2393	3259	867	0.088811995
203	A	JN798585	A82	2209	3075	867	0.088811995
204	A	MH828515	A82	1699	2565	867	0.027681661
205	A	LC428136	A82	1	867	867	0.0911188
206	A	LC428149	A82	1	867	867	0.089965398
207	A	LC428139	A88	1	870	870	0.088505747
208	A	LC428138	A88	1	870	870	0.087356322

209	A	NC_001617	A89	2341	3234	894	0
210	A	MW587058	A89	2341	3216	876	0.086757991
211	A	JQ837716	A89	2340	3215	876	0.076484018
212	A	JQ837719	A89	2266	3141	876	0.075342466
213	A	MW587076	A94	2314	3180	867	0.084198385
214	A	MW587054	A94	2314	3180	867	0.085351788
215	A	MW587078	A94	2229	3095	867	0.084198385
216	A	MW587083	A96	2249	3115	867	0.096885813
217	A	GQ415052	A101	2320	3186	867	0.061130334
218	A	MK989735	A101	2312	3178	867	0.070357555
219	A	JQ245965	A101	2120	2986	867	0.006920415
220	A	MN488754	A101	<1	864	>864	0.041666667
221	A	MN488753	A101	<1	864	>864	0.032407407
222	A	LC428142	A101	1	867	867	0.070357555
223	A	JQ747749	A103	2234	3124	891	0.067340067
224	A	JQ994499	A103	2059	2949	891	0.01010101
225	A	MH685681	A104	2240	3092	853	0.01992966
226	A	JX193797	A104	2173	3027	855	0
227	A	JX074047	A104	2150	3004	855	0
228	A	JN562727	A104	2097	2951	855	0
229	A	JN614995	A105	2249	3112	864	0
230	A	JN990699	A105	2090	2953	864	0.016203704
231	A	JX025555	A106	2198	3094	897	0
232	A	KP737114	A109	172	1041	870	0
233	B	MK989744	B3	2176	3044	869	0.098914355
234	B	JF285331	B3	2303	3171	869	0.101265823
235	B	JF285330	B3	2303	3171	869	0.102416571
236	B	JF285329	B3	2303	3171	869	0.102416571
237	B	MN212904	B4	2310	3173	864	0.094907407
238	B	MH828512	B4	1696	2559	864	0.092592593
239	B	JN798573	B4	2032	2895	864	0.09375
240	B	OM001469	B6	2284	3156	873	0.086206897
241	B	OM001456	B6	2284	3156	873	0.085057471
242	B	OM001447	B6	2284	3156	873	0.088505747
243	B	OM001435	B6	2284	3156	873	0.082758621
244	B	OM001425	B6	2284	3156	873	0.081609195
245	B	OM001417	B6	2284	3156	873	0.085057471
246	B	OM001409	B6	2284	3156	873	0.082758621
247	B	OM001402	B6	2284	3156	873	0.089655172
248	B	OM001393	B6	2284	3156	873	0.088505747
249	B	OM001381	B6	2284	3156	873	0.087356322
250	B	OM001367	B6	2284	3156	873	0.088505747
251	B	OL961545	B6	2284	3156	873	0.085057471
252	B	OL961541	B6	2284	3156	873	0.088505747

253	B	OL961538	B6	2284	3156	873	0.085057471	
254	B	OL961522	B6	2284	3156	873	0.088505747	
255	B	MZ667416	B6	2321	3190	870	0.085351788	
256	B	LC495296	B6	2350	3222	873	0.085057471	
257	B	JX193795	B6	1944	2813	870	0.08189158	
258	B	JQ747748	B6	2043	2912	870	0.078431373	
259	B	JQ747745	B6	2127	2996	870	0.079584775	
260	B	JN815243	B6	1903	2772	870	0.085351788	
261	B	JN562723	B6	2037	2906	870	0.084198385	
262	B	MN488777	B14	1	>811	>811	0.10974106	
263	B	MW587087	B26	2327	3187	861	0.102206736	
264	B	JF285309	B27	2163	3023	861	0.084785134	
265	B	JF285308	B27	2163	3023	861	0.084785134	
266	B	MK989746	B37	2235	3098	864	0.077546296	
267	B	KX348030	B37	2312	3175	864	0.078703704	
268	B	OL961520	B42	2312	3175	864	0.09837963	
269	B	MN488778	B42	1	864	864	0.105324074	
270	B	MN306037	B42	2328	3191	864	0.109953704	
271	B	KX433168	B42	1752	2621	870	0.091435185	
272	B	JN562724	B42	2255	3118	864	0.087962963	
273	B	JF781507	B42	2181	3044	864	0.096064815	
274	B	JF781498	B42	2062	2925	864	0.09375	
275	B	GU568096	B42	2329	3192	864	0.091435185	
276	B	MK989745	B48	2223	3104	882	0.111880046	
277	B	MK989741	B48	2231	3112	882	0.111111111	
278	B	KX348032	B48	2226	3107	882	0.109977324	
279	B	KX348029	B48	2228	3109	882	0.109977324	
280	B	JN990698	B48	2161	3042	882	0.107709751	
281	B	JQ245970	B69	2141	3019	879	0.084186576	
282	B	JN562721	B69	1901	2779	879	0.084186576	
283	B	HQ123445	B69	1702	2580	879	0.073947668	
284	B	MW587080	B70	2345	3232	888	0.117117117	
285	B	MH828513	B70	1702	2589	888	0.115990991	
286	B	JX074054	B70	2196	3083	888	0.116225547	
287	B	JQ245974	B70	2256	3143	888	0.115990991	
288	B	JN990706	B70	2346	3239	894	0.111764706	
289	B	MW587091	B70	2322	3209	888	0.130630631	yes, but *p*-distance is located near the threshold of typing and there is no significant phylogenetic difference, so it is not considered as a novel type
290	B	MK989743	B72	2345	3211	867	0.098039216	
291	B	KF958309	B72	2333	3199	867	0.092272203	
292	B	JQ245969	B72	2040	2906	867	0.0911188	

293	B	JN798562	B72	2152	3018	867	0.095732411	
294	B	JN614997	B72	2228	3094	867	0.092272203	
295	B	JN562726	B72	2049	2915	867	0.089965398	
296	B	MK989747	B79	2311	3174	864	0.081018519	
297	B	JN990701	B83	2049	2915	867	0.088811995	
298	B	KX433159	B84	1780	2646	867	0.096399535	
299	B	KX351793	B84	1774	2640	867	0.099883856	
300	B	JX074048	B84	2194	3054	861	0.088410992	
301	B	JQ837723	B84	2073	2933	861	0.085946574	
302	B	JN798588	B84	2015	2875	861	0.09335443	
303	B	JN614991	B84	2010	2870	861	0.085946574	
304	B	JN541271	B84	2122	2982	861	0.091753775	
305	B	JF781502	B84	2102	2962	861	0.087108014	
306	B	JF781499	B84	2010	2870	861	0.087108014	
307	B	MH828514	B86	1702	2565	864	0.005787037	
308	B	MW587052	B86	2331	3194	864	0.134259259	yes, but *p*-distance is located near the threshold of typing and there is no significant phylogenetic difference, so it is not considered as a novel type
309	B	MW587086	B93	2256	3116	861	0.085946574	
310	B	MW587077	B97	2321	3181	861	0.109175377	
311	B	MW587055	B97	2257	3117	861	0.10719323	
312	B	MK989742	B100	2122	2985	864	0.050925926	
313	B	JX074052	B101	1925	2791	867	0.01622248	
314	B	JF781501	B101	2144	3007	864	0.017361111	
315	B	MW587088	B103	2422	3285	864	0.025462963	
316	B	JQ994497	B103	2012	2875	864	0	
317	B	JQ837721	B103	2046	2909	864	0	
318	B	JQ837717	B103	2041	2904	864	0.015046296	
319	B	JQ245972	B103	2010	2873	864	0.015046296	
320	B	JN815239	B103	2180	3052	873	0.031890661	
321	B	JN798572	B103	2179	3042	864	0.018518519	
322	B	KF958308	B104	2326	3204	879	0.065984073	
323	B	JF781506	B104	2078	2962	885	0.067058824	
324	B	KP736667	B105	253	1119	867	0.00461361	
325	B	KP736666	B105	175	1041	867	0	
326	B	KP736637	B106	210	1097	888	0.006756757	
327	B	KP736636	B106	211	1098	888	0	
328	B	MH899592	B107	2093	2953	861	0.224157956(from B97)	yes, a novel type RVB
329	C	OM001407	C1	2180	3004	825	0.093333333	
330	C	OM001354	C1	2180	3004	825	0.08969697	
331	C	HQ123443	C1	1696	2523	828	0.032608696	

332	C	LC004880	C1	1495	>2310	>816	0.050245098
333	C	LC004879	C1	1495	>2310	>816	0.050245098
334	C	LC004782	C1	1495	>2310	>816	0.045343137
335	C	HM236916	C1	1	>822	>822	0.03163017
336	C	HM236919	C1	1	>816	>816	0.031862745
337	C	HM236915	C1	1	>816	>816	0.030637255
338	C	JQ245968	C2	2309	3121	813	0.046740467
339	C	KF958310	C2	2309	3121	813	0.018450185
340	C	MK989755	C2	2308	3120	813	0.09102091
341	C	MK989752	C2	2175	2987	813	0.089790898
342	C	JN837695	C2	2054	2866	813	0.03198032
343	C	JX025557	C2	1891	2703	813	0.019680197
344	C	JN990703	C2	1905	2717	813	0.018450185
345	C	JN815248	C2	1897	2709	813	0.018450185
346	C	LC004888	C2	1498	>2310	>813	0.046740467
347	C	LC004884	C2	1498	>2310	>813	0.034440344
348	C	LC004860	C2	1498	>2310	>813	0.034440344
349	C	LC004850	C2	1498	>2310	>813	0.034440344
350	C	LC004845	C2	1498	>2310	>813	0.035670357
351	C	LC004844	C2	1498	>2310	>813	0.038130381
352	C	LC004843	C2	1498	2310	813	0.034440344
353	C	LC004824	C2	1498	>2310	>813	0.036900369
354	C	LC004798	C2	1498	>2310	>813	0.040590406
355	C	LC004788	C2	1498	>2310	>813	0.038130381
356	C	LC004778	C2	1498	>2310	>813	0.036900369
357	C	MN488789	C2	1	813	813	0.09102091
358	C	LC428167	C2	1	813	813	0.093480935
359	C	JN798567	C3	1951	2775	825	0.024242424
360	C	JN990700	C3	1896	2720	825	0.037575758
361	C	LC004834	C3	1489	>2313	>825	0.04969697
362	C	LC004816	C3	1489	>2313	>825	0.050909091
363	C	HM236917	C3	1	825	825	0.035151515
364	C	NC_009996	C4	2305	3126	822	0
365	C	JF781509	C4	1761	2582	822	0.015815085
366	C	KY369882	C5	2289	3113	825	0.054545455
367	C	KY369880	C5	2289	3113	825	0.04969697
368	C	LC004901	C5	1510	>2334	>825	0.026666667
369	C	LC004900	C5	1510	>2334	>825	0.027878788
370	C	LC004897	C5	1510	>2334	>825	0.026666667
371	C	LC004896	C5	1510	>2334	>825	0.027878788
372	C	LC004895	C5	1510	>2334	>825	0.026666667
373	C	LC004864	C5	1510	>2334	>825	0.026666667
374	C	LC004838	C5	1510	>2334	>825	0.04
375	C	LC004799	C5	1510	>2334	>825	0.029090909

376	C	JF317016	C6	2298	3122	825	0.036363636	
377	C	JN990702	C6	2297	3121	825	0.037575758	
378	C	KF734978	C6	2298	3122	825	0.037575758	
379	C	MW587068	C6	2220	3035	816	0.075980392	
380	C	MN306023	C6	2171	2995	825	0.064242424	
381	C	JN815245	C6	1856	2680	825	0.052121212	
382	C	MH685704	C6	2167	2991	825	0.043636364	
383	C	MH685706	C6	2109	2933	825	0.043636364	
384	C	LC004871	C6	1486	>2310	>825	0.04969697	
385	C	LC004869	C6	1486	>2310	>825	0.050909091	
386	C	LC004835	C6	1486	>2310	>825	0.060606061	
387	C	LC004830	C6	1486	>2310	>825	0.052121212	
388	C	LC004818	C6	1486	>2310	>825	0.042424242	
389	C	LC004813	C6	1486	>2310	>825	0.055757576	
390	C	LC004812	C6	1486	>2310	>825	0.056969697	
391	C	HM236953	C6	1	825	825	0.032727273	
392	C	HM236933	C6	1	825	825	0.032727273	
393	C	HM236932	C6	1	825	825	0.04	
394	C	HM236910	C6	1	825	825	0.037575758	
395	C	HM236909	C6	1	825	825	0.033939394	
396	C	LC428166	C6	1	825	825	0.060606061	
397	C	LC428165	C6	1	825	825	0.060606061	
398	C	LC428164	C6	1	825	825	0.060606061	
399	C	HM236937	C6	1	825	825	0.036363636	
400	C	HM236913	C6	1	825	825	0.038787879	
401	C	HM236900	C6	1	825	825	0.031515152	
402	C	HM236899	C6	1	825	825	0.033939394	
403	C	EU935601	C6	1	825	825	0.058181818	
404	C	MW587059	C7	2297	3112	816	0.090686275	
405	C	OM001451	C7	2263	3078	816	0.085784314	
406	C	OM001438	C7	2263	3078	816	0.087009804	
407	C	OM001408	C7	2246	3061	816	0.089460784	
408	C	JN837689	C7	2227	3042	816	0.019607843	
409	C	JN798559	C7	1915	2730	816	0.034313725	
410	C	JX025556	C7	1829	2644	816	0.034313725	
411	C	JN798570	C7	1933	2748	816	0.068627451	
412	C	JQ994495	C7	1992	2807	816	0.069498069	
413	C	HM236920	C7	1	816	816	0.028186275	
414	C	KY189320	C8	2287	3132	846	0.134751773	yes, but *p*-distance is located near the threshold of typing and there is no significant phylogenetic difference, so it is not considered as a novel type
415	C	KY189316	C8	2275	3120	846	0.100472813	

416	C	JQ245964	C8	1957	2784	828	0.130434783	yes, but *p*-distance is located near the threshold of typing and there is no significant phylogenetic difference, so it is not considered as a novel type
417	C	JQ245973	C8	2032	2859	828	0.130434783	yes, but *p*-distance is located near the threshold of typing and there is no significant phylogenetic difference, so it is not considered as a novel type
418	C	LC004877	C8	1495	>2322	>828	0.078502415	
419	C	LC004872	C8	1495	>2322	>828	0.074879227	
420	C	LC428175	C9	2290	3111	822	0.055147059	
421	C	MH828517	C9	1693	2514	822	0.041666667	
422	C	LC004899	C9	1492	>2307	>816	0.026960784	
423	C	LC004868	C9	1492	>2307	>816	0.025735294	
424	C	LC004841	C9	1492	>2307	>816	0.044117647	
425	C	LC004837	C9	1492	>2307	>816	0.042892157	
426	C	LC004825	C9	1492	>2307	>816	0.026960784	
427	C	LC004814	C9	1492	>2307	>816	0.040441176	
428	C	MH685698	C11	2239	3057	819	0.026862027	
429	C	LC004910	C11	1507	>2325	>819	0.065934066	
430	C	LC004856	C11	1507	>2325	>819	0.062271062	
431	C	LC004839	C11	1507	>2325	>819	0.042735043	
432	C	JF317017	C12	2330	3160	831	0.025270758	
433	C	KP890663	C12	2325	3161	837	0.064748201	
434	C	KP242035	C12	2238	3068	831	0.032490975	
435	C	LC004866	C12	1507	>2343	>837	0.021582734	
436	C	LC004855	C12	1507	>2343	>837	0.056354916	
437	C	LC004849	C12	1507	>2343	>837	0.026378897	
438	C	LC004820	C12	1507	>2343	>837	0.017985612	
439	C	LC004794	C12	1507	>2343	>837	0.026378897	
440	C	LC004792	C12	1507	>2343	>837	0.022781775	
441	C	LC004790	C12	1507	>2343	>837	0.022781775	
442	C	LC428171	C12	1	834	834	0.063549161	
443	C	LC428169	C12	1	834	834	0.039568345	
444	C	HM236962	C12	1	834	834	0.011990408	
445	C	HM236958	C12	1	834	834	0	
446	C	HM236959	C12	1	831	831	0.013237064	
447	C	OL961524	C13	2264	3079	816	0.052696078	
448	C	OL961531	C13	2268	3083	816	0.050245098	
449	C	MZ268695	C13	2266	3081	816	0.050245098	
450	C	LC004898	C13	1492	>2307	>816	0.009803922	
451	C	LC004893	C13	1492	>2307	>816	0.009803922	

452	C	LC004852	C13	1492	>2307	>816	0.029411765
453	C	MH685707	C14	1413	2228	816	0.107843137
454	C	LC004781	C14	1498	>2313	>816	0.106617647
455	C	HM236911	C14	1	816	816	0
456	C	JF317014	C15	2310	3146	837	0.051558753
457	C	MN369033	C15	2309	3145	837	0.082733813
458	C	KY348864	C15	2298	3134	837	0.03117506
459	C	MN306035	C15	2231	3067	837	0.079136691
460	C	JN837688	C15	2230	3057	828	0.081212121
461	C	MN306026	C15	1940	2776	837	0.087529976
462	C	MT641442	C15	1777	2613	837	0.047961631
463	C	LC004891	C15	1501	>2337	>837	0.021582734
464	C	LC004874	C15	1501	>2337	>837	0.026378897
465	C	LC004873	C15	1501	>2337	>837	0.028776978
466	C	LC004851	C15	1501	>2337	>837	0.037170264
467	C	LC004832	C15	1501	>2337	>837	0.051558753
468	C	LC004829	C15	1501	>2337	>837	0.034772182
469	C	HM236963	C15	<1	834	>834	0
470	C	KR997882	C16	2148	2972	825	0.055961071
471	C	LC004894	C16	1492	>2316	>825	0.008515815
472	C	LC004892	C16	1492	>2316	>825	0.008515815
473	C	LC004827	C16	1492	>2316	>825	0.055961071
474	C	HM236944	C16	<1	822	>822	0
475	C	MZ268701	C17	2277	3093	817	0.05630355
476	C	MZ667421	C17	2271	3113	843	0.02189781
477	C	JN815244	C17	2202	3029	828	0.00875
478	C	JN815240	C17	2100	2924	825	0.018248175
479	C	JQ837720	C17	1939	2766	828	0.041975309
480	C	LC004909	C17	1498	>2322	>825	0.025547445
481	C	LC004804	C17	1498	>2322	>825	0.03406326
482	C	LC428177	C18	2290	3135	846	0.039285714
483	C	LC004821	C18	1498	>2343	>846	0.086904762
484	C	LC004775	C18	1498	>2343	>846	0.016666667
485	C	LC004774	C18	1498	>2343	>846	0.01547619
486	C	LC428170	C18	<1	843	>843	0.039285714
487	C	HM236956	C18	<1	843	>843	0.001190476
488	C	HM236950	C18	<1	843	>843	0.002380952
489	C	HM236948	C18	<1	843	>843	0.001190476
490	C	HM236955	C18	<1	840	>840	0.001190476
491	C	HM236918	C18	<1	840	>840	0
492	C	MW587060	C19	2326	3180	855	0.07030303
493	C	MK989748	C19	2322	3122	801	0.064918851
494	C	MH685694	C19	2312	3135	824	0.078883495
495	C	LC004854	C19	1525	>2349	>825	0.077575758

496	C	LC004853	C19	1525	>2349	>825	0.078787879
497	C	LC004817	C19	1525	>2349	>825	0.078787879
498	C	LC004815	C19	1525	>2349	>825	0.077575758
499	C	LC004811	C19	1525	>2349	>825	0.078787879
500	C	MK989753	C20	2318	3137	820	0.087804878
501	C	MK989751	C21	2363	3178	816	0.049019608
502	C	HM236930	C21	1	816	816	0.009803922
503	C	KJ675507	C22	2301	3116	816	0.036764706
504	C	JN621242	C22	2211	3026	816	0.036764706
505	C	HM236951	C22	1	816	816	0
506	C	HM236905	C22	1	816	816	0
507	C	KJ675506	C23	2326	3150	825	0.084043849
508	C	LC004842	C23	1507	>2334	>828	0.092570037
509	C	LC004836	C23	1507	>2334	>828	0.093788063
510	C	LC004777	C23	1507	>2334	>828	0.085470085
511	C	LC004776	C23	1507	>2334	>828	0.087912088
512	C	LC428168	C23	<1	825	>825	0.097442144
513	C	HM236946	C23	<1	825	>825	0.080586081
514	C	MG148341	C24	2275	3099	825	0.064476886
515	C	LC004867	C24	1498	>2322	>825	0.060827251
516	C	LC004846	C24	1498	>2322	>825	0.063260341
517	C	JF317013	C25	2312	3136	825	0.02238806
518	C	MN369030	C25	2183	3007	825	0.039800995
519	C	JN837685	C25	2109	2933	825	0.082089552
520	C	HQ123440	C25	1702	2526	825	0.074626866
521	C	LC004905	C25	1501	>2325	>825	0.023631841
522	C	LC004882	C25	1501	>2325	>825	0.029850746
523	C	LC004833	C25	1501	>2325	>825	0.036069652
524	C	LC004797	C25	1501	>2325	>825	0.032338308
525	C	KP890664	C26	2336	3157	822	0.068126521
526	C	MH685697	C26	2241	3062	822	0.075425791
527	C	JX193796	C26	1927	2748	822	0.037712895
528	C	LC004876	C26	1495	>2316	>822	0.062043796
529	C	LC004848	C26	1495	>2316	>822	0.060827251
530	C	LC004787	C26	1495	>2316	>822	0.064476886
531	C	HM236957	C26	1	822	822	0.00243309
532	C	MN488791	C26	<1	816	>816	0.067401961
533	C	MH828521	C27	1714	2526	813	0.035670357
534	C	HM236914	C27	1	812	812	0.020935961
535	C	HM236907	C27	1	813	813	0
536	C	HM236928	C27	1	813	813	0.01599016
537	C	MZ447875	C28	2218	3063	846	0.067388688
538	C	JN798569	C28	1951	2784	834	0.033939394
539	C	LC004907	C28	1498	>2325	>828	0.055757576

540	C	LC004810	C28	1498	>2325	>828	0.063030303
541	C	LC004809	C28	1498	>2325	>828	0.063030303
542	C	LC004808	C28	1498	>2325	>828	0.064242424
543	C	HM236949	C29	1	816	816	0
544	C	MK989754	C30	933	1754	822	0.053921569
545	C	LC004870	C30	1501	>2322	>822	0.049019608
546	C	LC004784	C30	1501	>2322	>822	0.050245098
547	C	HM236968	C30	<1	816	>816	0
548	C	MW587053	C31	2176	3020	845	0.076537014
549	C	KY189319	C31	2161	3005	845	0.079046424
550	C	LC004840	C31	1495	>2322	>828	0.051442911
551	C	KY348786	C32	2220	3038	819	0.072303922
552	C	JN798581	C32	2269	3087	819	0.022058824
553	C	JQ994498	C32	2216	3034	819	0.014705882
554	C	MT641372	C32	1765	2583	819	0.077205882
555	C	HM236943	C32	1	816	816	0.011029412
556	C	HM236941	C32	1	816	816	0.011029412
557	C	HM236924	C32	1	816	816	0.014705882
558	C	HM236921	C32	1	816	816	0.011029412
559	C	HM236902	C32	1	816	816	0.011029412
560	C	HM236942	C32	1	816	816	0.012254902
561	C	HM236940	C32	1	816	816	0.011029412
562	C	HM236938	C32	1	816	816	0.011029412
563	C	HM236922	C32	1	813	813	0.009840098
564	C	OM001346	C33	2284	3111	828	0.057971014
565	C	HM236965	C33	1	816	816	0.020833333
566	C	MH685696	C34	2255	3081	827	0.091898428
567	C	KM486097	C34	1705	2531	827	0.089480048
568	C	JF436926	C34	1218	2048	831	0.026570048
569	C	KX348031	C35	2099	2914	816	0.078431373
570	C	LC004906	C35	1492	>2307	>816	0.074754902
571	C	MZ438010	C36	2237	3052	816	0.019753086
572	C	JN541267	C36	2225	3040	816	0.104938272
573	C	MH828520	C36	1696	2511	816	0.114814815
574	C	LC004885	C36	1495	>2310	>816	0.107407407
575	C	LC004796	C36	1495	>2310	>816	0.10617284
576	C	LC004908	C37	1495	>2307	>813	0.04679803
577	C	JN837691	C38	1400	2224	825	0.034355828
578	C	LC004863	C38	1510	>2334	>825	0.040490798
579	C	JF416322	C38	1	>815	>815	0
580	C	JN205461	C39	2307	3125	819	0
581	C	LC004847	C39	1489	>2307	>819	0.031746032
582	C	LC004828	C39	1489	>2307	>819	0.030525031
583	C	JF416306	C39	1	>818	>818	0.025672372

584	C	JQ245963	C40	2138	2950	813	0.068880689
585	C	JF781505	C40	2149	2961	813	0.065190652
586	C	JN815251	C40	2085	2897	813	0
587	C	LC004831	C40	1498	>2310	>813	0.028290283
588	C	LC004801	C40	1498	>2310	>813	0.03198032
589	C	LC428174	C40	1	813	813	0.033210332
590	C	JF416315	C40	1	>812	>812	0.068965517
591	C	JF416313	C40	1	>812	>812	0.066502463
592	C	JF416312	C40	1	>812	>812	0.070197044
593	C	KF958311	C41	2327	3148	822	0
594	C	KY189321	C41	2302	3123	822	0.090024331
595	C	JN798565	C41	2214	3035	822	0
596	C	MK989750	C41	1879	2700	822	0.088807786
597	C	LC004875	C41	1501	>2322	>822	0.081508516
598	C	LC004826	C41	1501	>2322	>822	0.077858881
599	C	KJ675505	C42	2331	3155	825	0.041564792
600	C	MZ268666	C42	2310	3134	825	0.10391198
601	C	MH330336	C42	2317	3141	825	0.0599022
602	C	JQ994500	C42	2352	3179	828	0.079462103
603	C	MT641410	C42	1792	2616	825	0.102689487
604	C	LC004890	C42	1507	>2334	>828	0.078239609
605	C	LC004889	C42	1507	>2334	>828	0.078239609
606	C	LC004887	C42	1507	>2334	>828	0.078239609
607	C	LC004858	C42	1507	>2334	>828	0.078239609
608	C	LC004807	C42	1507	>2334	>828	0.079462103
609	C	LC004805	C42	1507	>2334	>828	0.091687042
610	C	LC004803	C42	1507	>2334	>828	0.083129584
611	C	LC004802	C42	1507	>2334	>828	0.085574572
612	C	JF416320	C42	1	>818	>818	0
613	C	JX074056	C43	2182	2997	816	0.078244275
614	C	JN815249	C43	2029	2865	837	0
615	C	JN837687	C43	1970	2785	816	0.026717557
616	C	LC004883	C43	1489	>2304	>816	0.083969466
617	C	LC004881	C43	1489	>2304	>816	0.078244275
618	C	LC004878	C43	1489	>2304	>816	0.076335878
619	C	LC004795	C43	1489	>2304	>816	0.080152672
620	C	LC004791	C43	1489	>2304	>816	0.078244275
621	C	LC004789	C43	1489	>2304	>816	0.080152672
622	C	JF416309	C43	1	>815	>815	0.072657744
623	C	JF416307	C43	1	>815	>815	0.059273423
624	C	OM001412	C44	2291	3124	834	0.056422569
625	C	OL961544	C44	2112	2945	834	0.049219688
626	C	OL961535	C44	2112	2945	834	0.048019208
627	C	LC004865	C44	1507	>2343	>837	0.020408163

628	C	JF416310	C44	1	>833	>833	0
629	C	JN837686	C45	2310	3128	819	0.03803681
630	C	KY624849	C45	2188	3006	819	0.060122699
631	C	LC004857	C45	1507	>2325	>819	0.046625767
632	C	MH828519	C46	1696	2511	816	0.047911548
633	C	MH828518	C46	1696	2511	816	0.047911548
634	C	LC004819	C46	1495	>2310	>816	0.011056511
635	C	JF519760	C47	25	837	813	0
636	C	MW679007	C48	2211	3026	816	0.074754902
637	C	LC004800	C48	1498	>2313	>816	0.022058824
638	C	JN798566	C49	2299	3120	822	0.030413625
639	C	JF907574	C49	1864	2685	822	0.015815085
640	C	JN798568	C49	1887	2708	822	0.02919708
641	C	JF946738	C49	25	846	822	0
642	C	JQ739202	C50	96	914	819	0.01953602
643	C	JX291115	C51	2341	3153	813	0.004920049
644	C	MH685695	C51	2259	3080	822	0.03406326
645	C	KP736724	C52	158	976	819	0
646	C	MK989756	C53	2247	3068	822	0
647	C	MK989758	C53	2352	3173	822	0.114355231
648	C	MK989749	C53	2341	3162	822	0.01459854
649	C	KY369879	C53	2309	3130	822	0.01703163
650	C	KU695562	C53	2321	3142	822	0.03406326
651	C	LC428176	C53	2299	3120	822	0.01946472
652	C	MK989759	C53	2214	3035	822	0.112531969
653	C	MH685705	C53	2157	2978	822	0.032846715
654	C	LC004904	C53	1495	>2316	>822	0.114355231
655	C	LC004903	C53	1495	>2316	>822	0.114355231
656	C	LC004786	C53	1495	>2316	>822	0.079075426
657	C	LC004785	C53	1495	>2316	>822	0.114355231
658	C	LC004783	C53	1495	>2316	>822	0.116788321
659	C	KP736726	C53	158	979	822	0.111922141
660	C	LC428172	C53	1	822	822	0.01946472
661	C	LC004859	C54	1507	>2322	>816	0.013480392
662	C	LC004822	C54	1507	>2322	>816	0.011029412
663	C	LC004780	C54	1507	>2322	>816	0.008578431
664	C	LC004779	C54	1507	>2322	>816	0.013480392
665	C	LC004773	C54	1507	>2322	>816	0.012254902
666	C	KR997885	C55	2136	2954	819	0
667	C	LC004862	C55	1492	>2316	>825	0.06004902
668	C	LC004861	C55	1492	>2316	>825	0.06004902
669	C	LC004823	C55	1492	>2316	>825	0.0625
670	C	LC004793	C55	1492	>2316	>825	0.06127451
671	C	MZ268682	C56	2298	3143	846	0.043735225

672	C	MW679009	C56	2299	3144	846	0.047281324	
673	C	LC004772	C56	1498	>2343	>846	0	
674	C	KP890662	C57	2301	3116	816	0	
675	C	LC004902	C58	1498	>2313	>816	0.224264706 （from C14）	yes, a novel type RVC
676	C	MN369031	C59	2292	3114	823	0.206388206 （from C55）	yes, a novel type RVC
677	C	MF775365	C59	2332	3156	825	0.202205882 （from C55）	yes, a novel type RVC
678	C	LC004886	C59	1492	>2316	>825	0.196078431 （from C55）	yes, a novel type RVC
679	C	LC004806	C59	1492	>2295	>804	0.190298507 （from C55）	yes, a novel type RVC
680	C	MK989757	C60	2331	3146	816	0.166030534 （from C43）	yes, a novel type RVC

Table5: GC content table of different serotypes of RV genome.

Species	Serotype	Number	GC content average	GC content maximum	GC content minimum	GC content standard deviation
RVA	1	277	0.3817	0.3837	0.3724	0.0021
	2	10	0.3885	0.3902	0.3871	0.0009
	7	6	0.3874	0.3927	0.3808	0.0050
	8	2	0.3931	0.3931	0.3931	0.0000
	9	5	0.3864	0.3876	0.3851	0.0009
	10	2	0.3908	0.3908	0.3908	0.0000
	11	15	0.3849	0.3860	0.3808	0.0015
	12	4	0.3955	0.3980	0.3933	0.0020
	13	9	0.3903	0.3915	0.3880	0.0013
	15	1	0.3825	0.3825	0.3825	
	16	6	0.3820	0.3850	0.3799	0.0019
	18	3	0.3853	0.3881	0.3803	0.0043
	19	2	0.3864	0.3901	0.3828	0.0052
	20	17	0.3910	0.3932	0.3836	0.0028
	21	16	0.3762	0.3828	0.3739	0.0027
	22	11	0.3963	0.3978	0.3940	0.0015
	23	50	0.3867	0.3880	0.3850	0.0005
	24	20	0.3818	0.3861	0.3780	0.0025
	25	32	0.3807	0.3829	0.3755	0.0014
	28	9	0.3868	0.3888	0.3848	0.0013
	29	16	0.3857	0.3888	0.3812	0.0022
	30	20	0.3869	0.3896	0.3855	0.0010
	31	14	0.3814	0.3835	0.3795	0.0013
	32	1	0.3778	0.3778	0.3778	
	33	3	0.3857	0.3892	0.3839	0.0030
	34	19	0.3848	0.3901	0.3795	0.0025
	36	4	0.3916	0.3939	0.3899	0.0017
	38	3	0.3832	0.3838	0.3821	0.0010
	39	43	0.3884	0.3910	0.3848	0.0013
	40	3	0.3827	0.3854	0.3785	0.0037
	41	2	0.3949	0.3971	0.3928	0.0030
	43	1	0.3803	0.3803	0.3803	
	45	4	0.3953	0.3978	0.3927	0.0021
	46	7	0.3887	0.3897	0.3862	0.0012
	47	7	0.3821	0.3851	0.3742	0.0038
	49	25	0.3905	0.3935	0.3834	0.0022
	50	1	0.3826	0.3826	0.3826	
	51	2	0.3893	0.3905	0.3880	0.0018
	53	5	0.3888	0.3919	0.3868	0.0020
	54	10	0.3846	0.3915	0.3757	0.0051

	55	1	0.3800	0.3800	0.3800	
	56	1	0.3789	0.3789	0.3789	
	57	1	0.3768	0.3768	0.3768	
	58	9	0.3871	0.3892	0.3853	0.0013
	59	9	0.3857	0.3873	0.3839	0.0012
	60	8	0.3837	0.3865	0.3790	0.0023
	61	11	0.3901	0.3911	0.3889	0.0008
	62	3	0.3752	0.3766	0.3732	0.0018
	63	4	0.3829	0.3869	0.3798	0.0031
	64	6	0.3952	0.3961	0.3945	0.0006
	65	3	0.3942	0.3956	0.3926	0.0015
	66	8	0.3846	0.3882	0.3823	0.0025
	67	18	0.3899	0.3919	0.3868	0.0011
	68	3	0.3869	0.3875	0.3863	0.0006
	71	1	0.3895	0.3895	0.3895	
	73	2	0.3937	0.3941	0.3933	0.0006
	74	1	0.3864	0.3864	0.3864	
	75	1	0.3835	0.3835	0.3835	
	76	2	0.3884	0.3887	0.3882	0.0004
	77	1	0.3765	0.3765	0.3765	
	78	42	0.3902	0.3938	0.3864	0.0013
	80	12	0.3902	0.3913	0.3869	0.0011
	81	4	0.3880	0.3894	0.3858	0.0016
	82	4	0.3898	0.3902	0.3895	0.0004
	85	4	0.3848	0.3855	0.3841	0.0006
	88	2	0.3901	0.3907	0.3895	0.0009
	89	10	0.3910	0.3932	0.3897	0.0013
	90	1	0.3796	0.3796	0.3796	
	94	8	0.3949	0.3980	0.3918	0.0021
	96	2	0.3906	0.3911	0.3901	0.0007
	100	3	0.3859	0.3864	0.3852	0.0007
	101	135	0.3934	0.3960	0.3891	0.0015
	103	3	0.3861	0.3866	0.3857	0.0004
	104	1	0.3751	0.3751	0.3751	
	105	4	0.3828	0.3838	0.3811	0.0012
	110	1	0.3871	0.3871	0.3871	
RVB	3	13	0.4025	0.4059	0.3994	0.0022
	4	5	0.3950	0.3966	0.3934	0.0012
	5	1	0.3894	0.3894	0.3894	
	6	88	0.4010	0.4047	0.3953	0.0015
	14	10	0.4067	0.4088	0.4057	0.0012
	17	1	0.4124	0.4124	0.4124	
	26	2	0.3905	0.3914	0.3897	0.0012
	27	34	0.3954	0.3979	0.3918	0.0011

	35	2	0.3983	0.3984	0.3983	0.0001
	37	3	0.3964	0.3969	0.3956	0.0007
	42	5	0.3959	0.4037	0.3807	0.0098
	48	2	0.4060	0.4096	0.4024	0.0051
	52	2	0.4139	0.4139	0.4138	0.0001
	69	1	0.4164	0.4164	0.4164	
	70	6	0.4100	0.4126	0.4069	0.0019
	72	5	0.4045	0.4063	0.4037	0.0010
	79	3	0.3976	0.4005	0.3951	0.0027
	83	2	0.3924	0.3925	0.3923	0.0002
	84	3	0.3905	0.3930	0.3882	0.0024
	86	5	0.3973	0.4013	0.3874	0.0057
	91	5	0.4102	0.4111	0.4082	0.0012
	92	1	0.4000	0.4000	0.4000	
	93	2	0.3951	0.3961	0.3942	0.0013
	97	4	0.3935	0.3976	0.3912	0.0028
	99	1	0.3862	0.3862	0.3862	
	100	4	0.3927	0.3929	0.3921	0.0004
	101	3	0.3882	0.3902	0.3871	0.0017
	102	1	0.4016	0.4016	0.4016	
	103	3	0.4006	0.4024	0.3989	0.0017
	104	4	0.4120	0.4144	0.4109	0.0016
	107	2	0.3907	0.3907	0.3907	0.0000
	1	16	0.4335	0.4352	0.4316	0.0011
	2	8	0.4370	0.4399	0.4319	0.0032
	3	38	0.4319	0.4345	0.4300	0.0009
	4	2	0.4279	0.4279	0.4279	0.0000
	5	3	0.4179	0.4188	0.4173	0.0008
	6	8	0.4299	0.4330	0.4274	0.0018
	7	7	0.4415	0.4430	0.4361	0.0026
	8	10	0.4283	0.4311	0.4259	0.0021
	9	4	0.4302	0.4307	0.4300	0.0003
	10	1	0.4388	0.4388	0.4388	
RVC	11	49	0.4152	0.4197	0.4110	0.0016
	12	2	0.4276	0.4296	0.4256	0.0028
	13	6	0.4306	0.4312	0.4299	0.0004
	15	29	0.4297	0.4328	0.4262	0.0016
	17	49	0.4242	0.4261	0.4225	0.0009
	19	4	0.4284	0.4320	0.4260	0.0028
	20	47	0.4239	0.4258	0.4217	0.0009
	21	1	0.4562	0.4562	0.4562	
	22	1	0.4269	0.4269	0.4269	
	23	2	0.4292	0.4321	0.4263	0.0041
	24	1	0.4311	0.4311	0.4311	

25	2	0.4397	0.4404	0.4390	0.0010
26	1	0.4305	0.4305	0.4305	
27	1	0.4128	0.4128	0.4128	
28	3	0.4281	0.4290	0.4267	0.0013
31	5	0.4346	0.4369	0.4334	0.0016
32	2	0.4274	0.4278	0.4271	0.0005
33	7	0.4016	0.4022	0.4010	0.0004
34	4	0.4220	0.4228	0.4211	0.0007
36	14	0.4322	0.4335	0.4257	0.0020
39	1	0.4245	0.4245	0.4245	
40	4	0.4386	0.4396	0.4374	0.0011
41	3	0.4312	0.4335	0.4280	0.0029
42	20	0.4263	0.4297	0.4233	0.0014
43	8	0.4322	0.4334	0.4312	0.0008
44	9	0.4256	0.4274	0.4246	0.0008
45	2	0.4284	0.4304	0.4265	0.0027
47	1	0.4307	0.4307	0.4307	
48	5	0.4007	0.4017	0.3991	0.0012
50	1	0.4208	0.4208	0.4208	
51	2	0.4386	0.4388	0.4384	0.0002
53	7	0.4368	0.4391	0.4348	0.0018
54	1	0.4325	0.4325	0.4325	
55	3	0.4493	0.4500	0.4488	0.0006
56	18	0.4275	0.4315	0.4252	0.0016
57	1	0.4376	0.4376	0.4376	
59	2	0.4411	0.4416	0.4405	0.0008
60	1	0.4271	0.4271	0.4271	

Supplementary Figures Caption

Figure 1: Identity comparison with existing RV sequences of the same type in the GenBank.

Figure 2: The alignments plot shows the VP1 AA multiple sequence alignment of A110, A108, A109, A16, and the sequences closely related to the whole genome phylogeny with A110.

Figure 3: RVA Time-scale MCC tree built using BEAST based on VP1 with non-collapsed branches. Each node is labeled with NCBI accession number, and collection time, and samples in this study are labeled by strain name.

Figure 4: RVB Time-scale MCC tree built using BEAST based on VP1 with non-collapsed branches. Each node is labeled with NCBI accession number, and collection time, and samples in this study are labeled by strain name.

Figure 5: RVC Time-scale MCC tree built using BEAST based on VP1 with non-collapsed branches. Each node is labeled with NCBI accession number, and collection time, and samples in this study are labeled by its strain name.

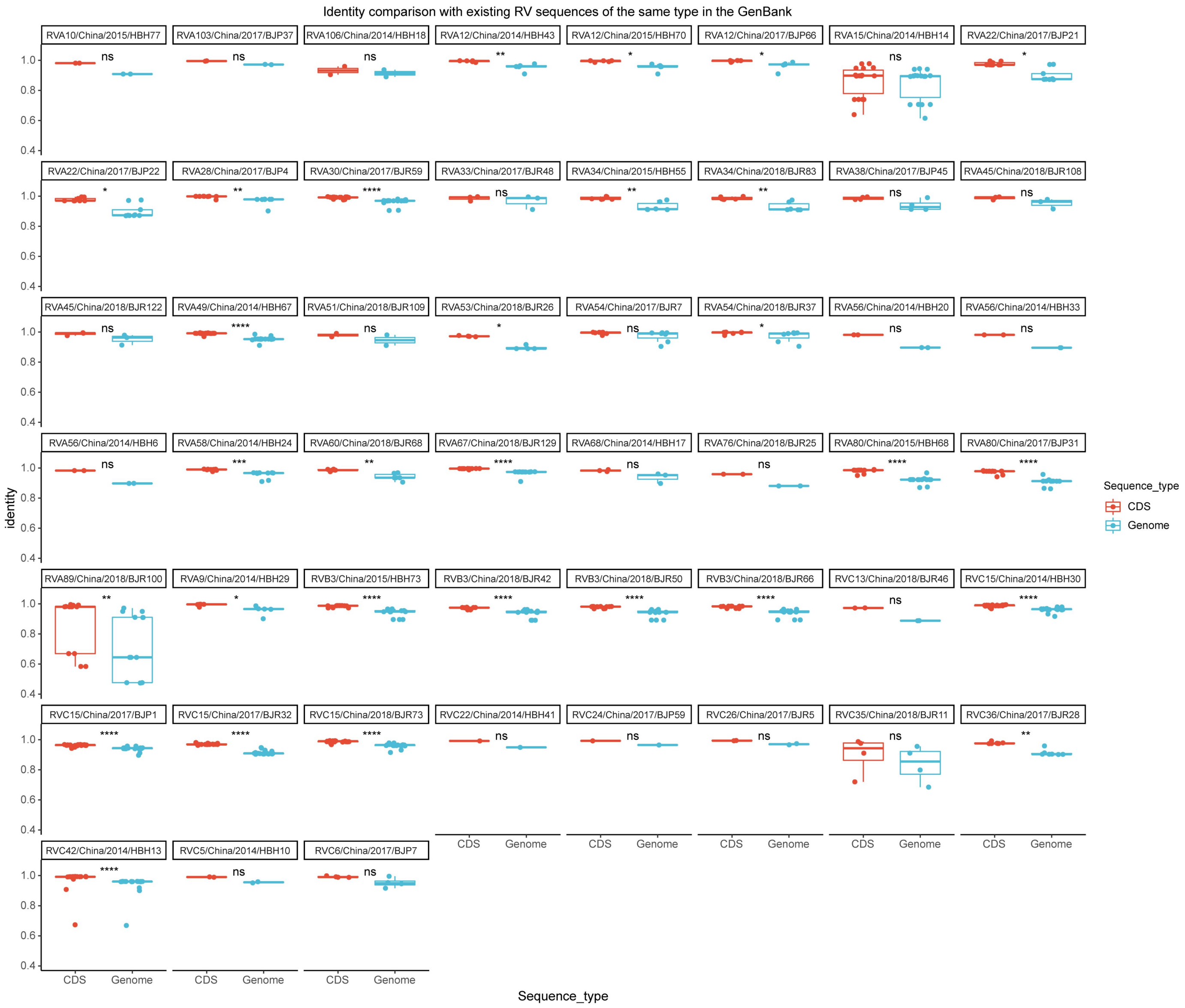

Supplementary Figure1

155×184mm

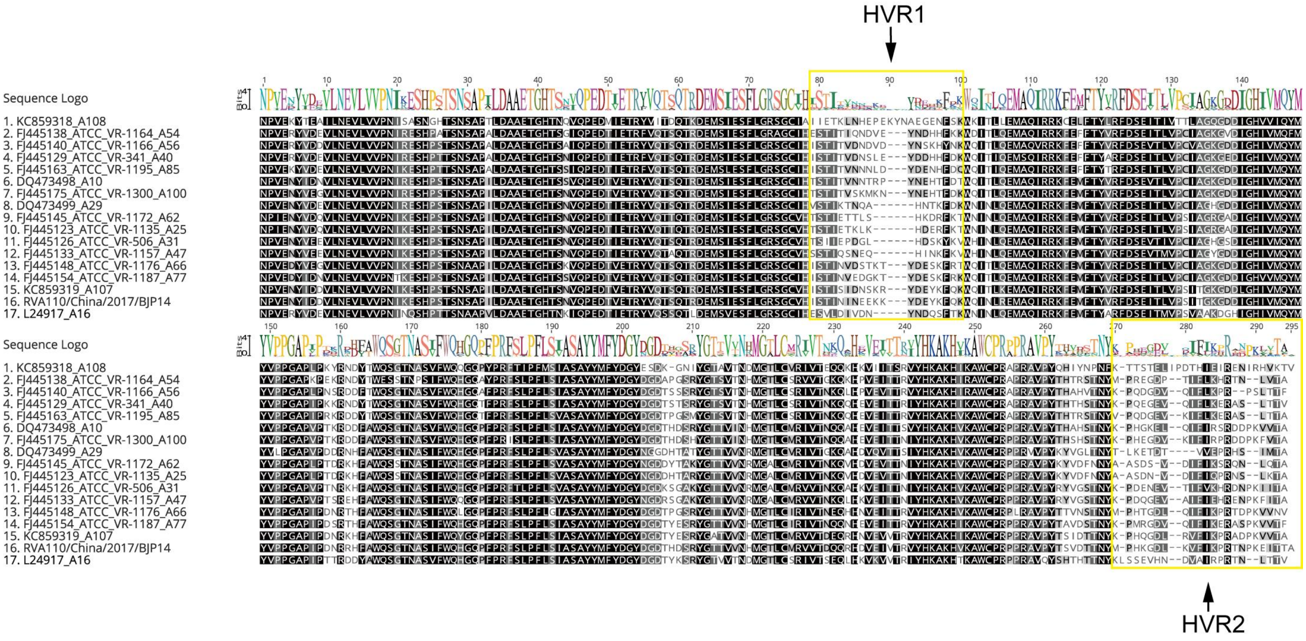

Supplementary Figure2

84×171mm

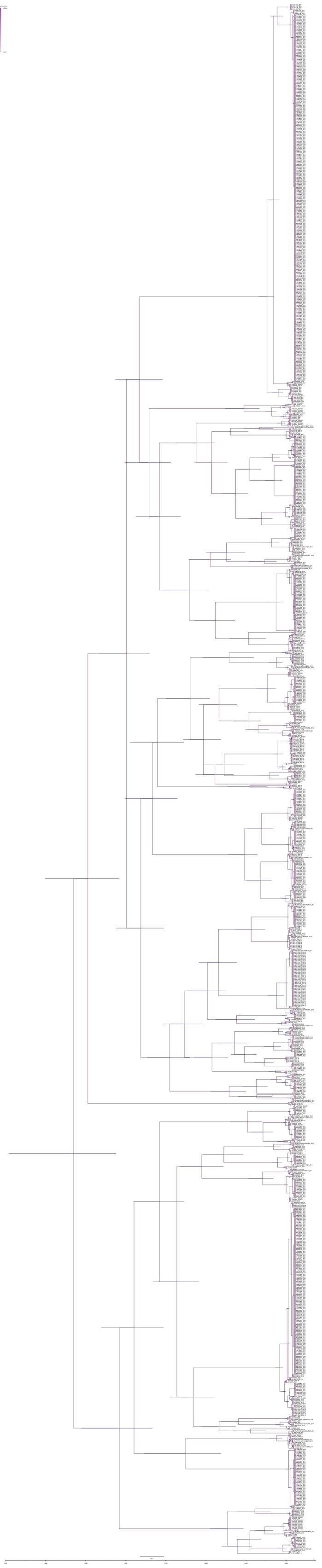

Supplementary Figure3

224×46mm

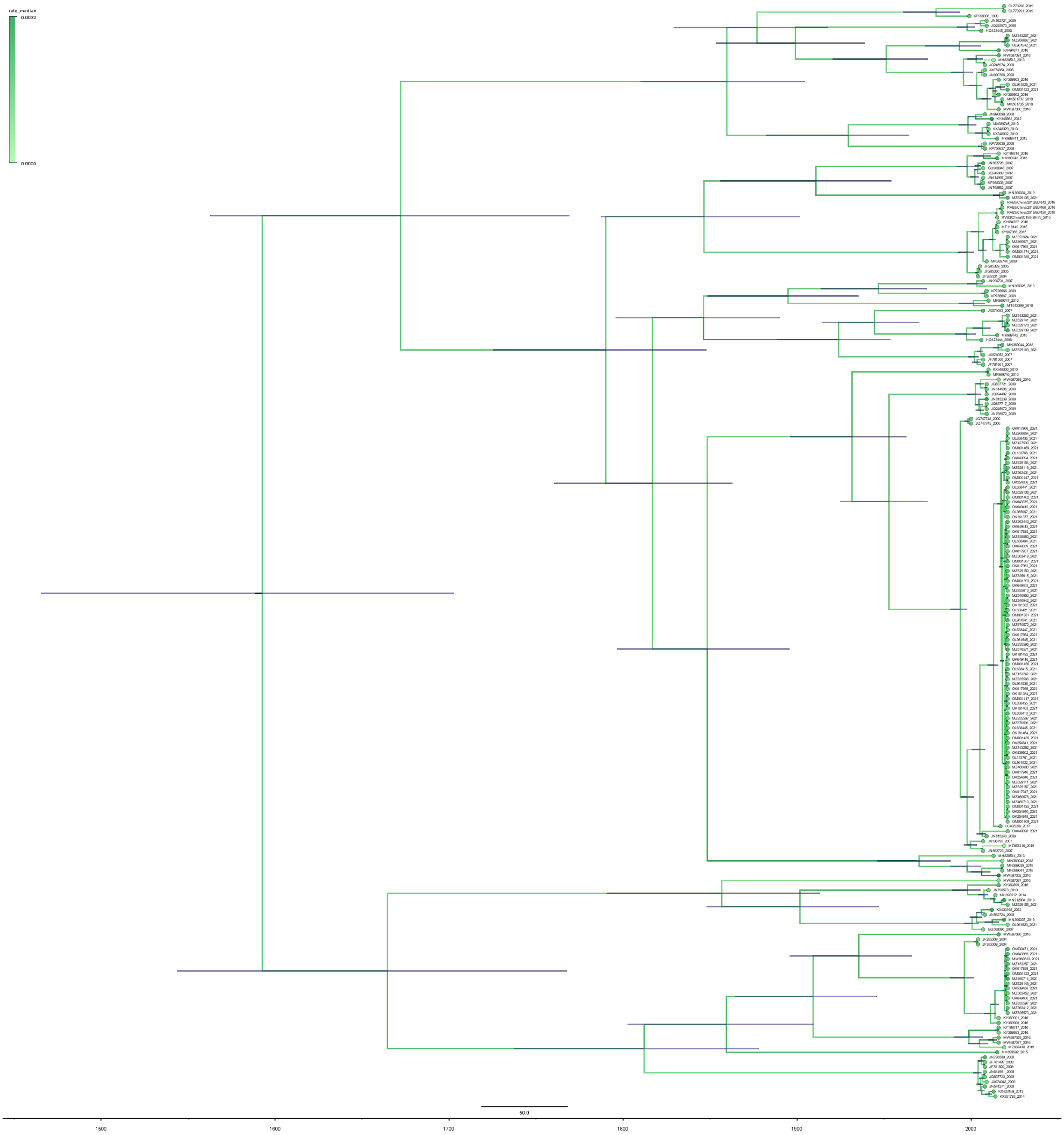

Supplementary Figure4

174×163mm

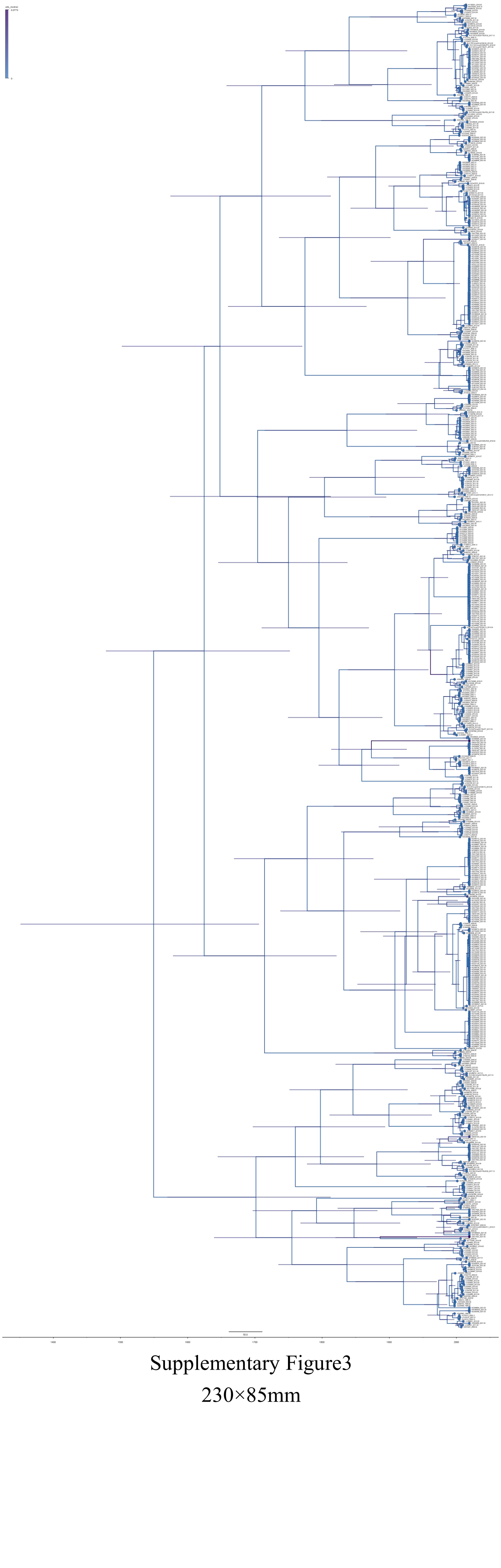

Supplementary Figure3

230×85mm
